# Three-Parameter Electric Dipole Moment Function for the CO Molecule

**DOI:** 10.1021/acs.jctc.4c00098

**Published:** 2024-05-22

**Authors:** Vladimír Špirko

**Affiliations:** Institute of Organic Chemistry and Biochemistry, p.r.i., Czech Academy of Sciences, Flemingovo nám. 2, 166 10 Prague 6, Czechia

## Abstract

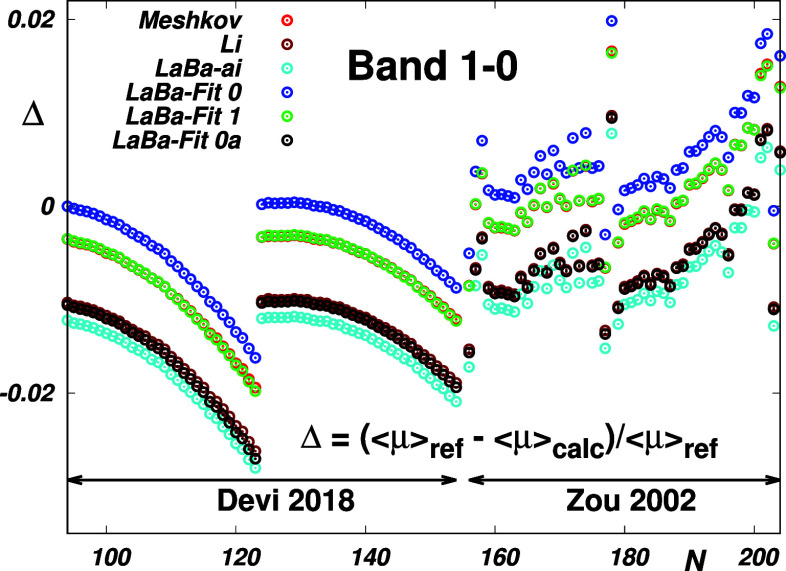

A global electric dipole moment function of the ground electronic state of carbon monoxide is constructed by morphing its best theoretical approximants from the literature to the best available experimental data within the framework of the reduced radial curve approach. The resulting functions coincide with their best many-parameter empirical counterparts so closely that they can be used as highly accurate three-parameter representations. Apparently, given the mathematical nature of the problem addressed, this approach can be applied equally well to all radial molecular functions that have similarly cumbersome shapes as the function probed. This means that the property characteristics of diatomic molecules can, in principle, be described with high precision even when as few as three pertinent experimental data points are accurately known. To date, no such functional approximants are available in the literature.

## 1 Introduction

**Figure 1:**
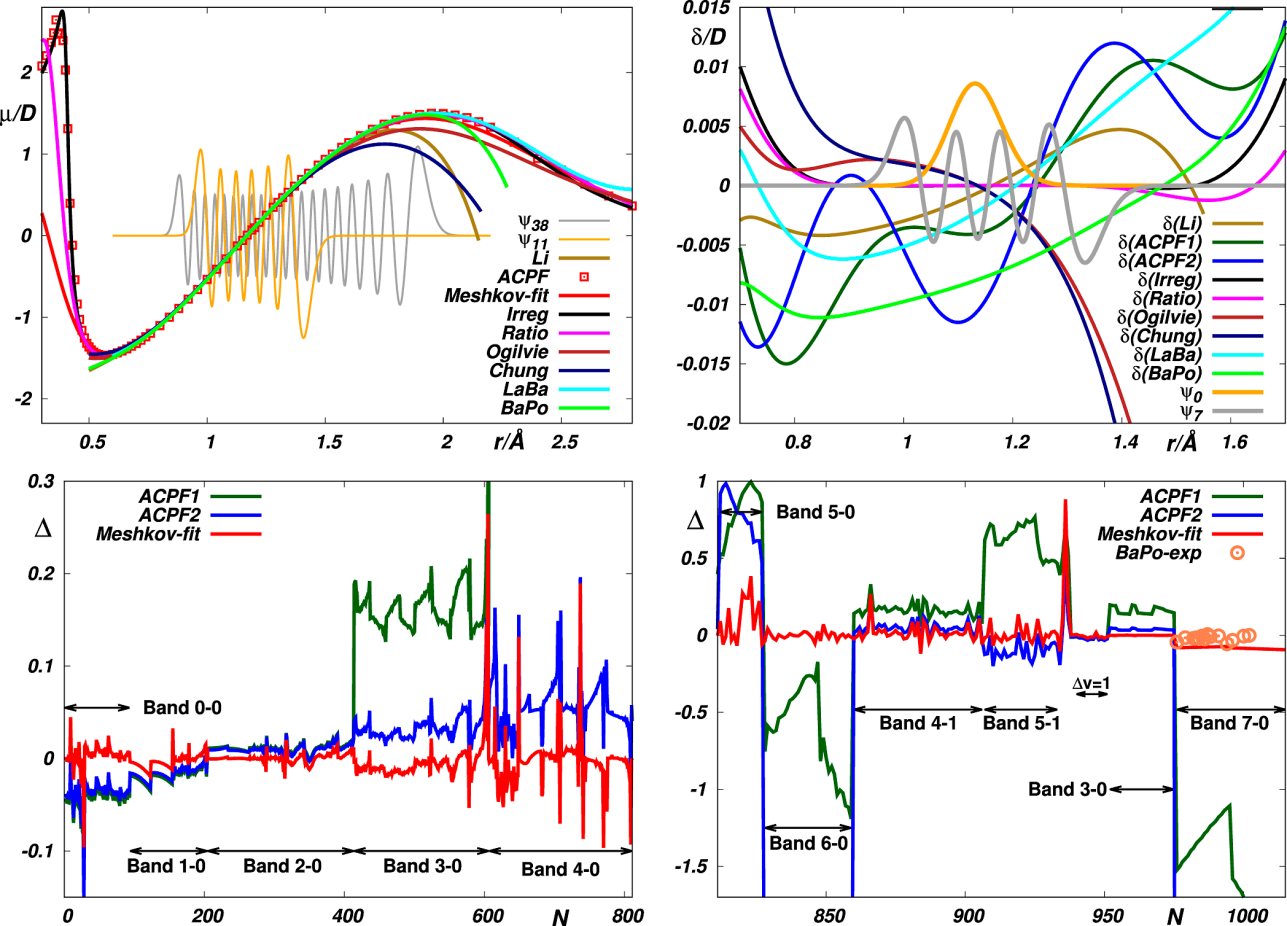
Top panels: Empirical *Li*,^1^*Meshkov-fit*,^4^*Irreg* and *Ratio*,^5^*Ogilvie*,^12^ and *Chung*(13) and theoretical *ACPF*,^4^*LaBa*,^14^ and *BaPo*(11) EDM functions μ of CO (left panel) and the deviations δ of these functions from the “reference” *Meshkov-fit* function (right panel). Discrete ab initio data were interpolated using exponentials (*ACPF1*, *LaBa*) and cubic splines (*ACPF2*). The wave functions ψ_38_ and ψ_11_ are for the highest vibrational states for which experimental ro-vibrational energies and intensities, respectively, are available. Lower panels: Reproduction of the CO reference data by the *ACPF* ab initio data of ref (4) interpolated in this study. Δ is defined by eq 7. *N* is a sequential number of the reference data (see Table S3 in Supporting Information).

Being the most common molecule in the universe after molecular hydrogen, carbon monoxide (CO) is one of the most important and promising probes of terrestrial, solar, and exoplanet atmospheres and of the interstellar medium (see, e.g., ref (1) and references therein). To probe these environments, accurate spectroscopic parameters, particularly line positions and line strengths, are required. To rationalize the usage of these parameters and enable their reliable prediction, accurate potential energy and electric dipole moment (EDM) functions are needed. Thanks to the recent efforts of Medvedev, Meshkov, and their co-workers,^2−5^ it appears that such functions can be constructed by fitting experimental and ab initio data while imposing certain mathematical restrictions guaranteeing their desirable behavior. It should be stressed, however, that, especially due to the fairly cumbersome shape of the probed EDM function (two maxima, two minima, and three intersections with an abscissa at *r* > 0, Figure 1), its construction from spectral data is strongly hampered by the number of necessary fitting parameters (e.g., 13 and 14 adjustable parameters in refs (4 and 5) respectively). Fortunately, however, thanks to the availability of highly accurate measured intensities for a broad range of vibration states, this fact, given the low flexibility of the elementary mathematical functions used, is not limiting. Actually, the functions discussed provide for an almost equivalent description of the experimental data currently available for all CO isotopomers, save for the “abnormal” 5-0 vibrational band.^4^ Unfortunately, a relative abundance of experimental data is more of an exception than a normal situation, which prohibits the robust determination of similar functions for many other molecular systems. As recently proposed in ref (6), one way to overcome this problem may rely on the homotopic morphing of purely theoretical functions within the framework of Jenč’s basically “three-parameter” reduced potential energy curve (RPC) scheme (see refs (7–9) and references therein). So far, this approach has been applied only to the dipole moment functions of the hydrogen halides HF, HCl, and HBr, which are all very simple in shape. The aim of this study is to explore the limits and possibilities of this approach with regard to functions that are more complex in shape, as faced, for example, in the case of CO. Carbon monoxide is one of the very few molecules for which subper-cent accurate line-intensity measurements have been made (see ref (10)). Moreover, its recently evaluated EDM function exhibits a physically correct shape (see ref (11)). For these reasons, the CO molecule appears to be an ideal candidate for such an exploration.

## 2 Theory

Quite generally, the reduced radial curve (RRC) approach consists of two steps: First, a given (reference) single-minimum radial function *F*^ref^ is used to generate its reduced form *f*(ρ), which is defined as follows
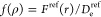
1where *D*_e_^ref^ is the depth of *F*^ref^(*r*) and the reduced variable ρ is related to *r* via the expression
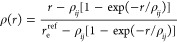
2Here, *r*_e_^ref^ is the distance for which *F*^ref^(*r*) acquires its minimum, and ρ_*ij*_ satisfies the transcendental equation
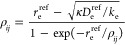
3where
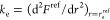
4κ being the “universal shape constant” (any value allowing for a numerically stable solution of the transcendental eq 2 for ρ_*ij*_).

In the second step, the reducing procedure is inverted by expressing *F*(*r*) as a function of *f*(ρ)
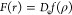
5with ρ defined by
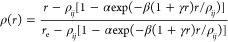
6and involving the a priori unknown parameters *D*_e_, *r*_e_, ρ_*ij*_, α, β, and γ, which are to be determined by fitting the experimental ro-vibrational data available (in the standard RRC scheme, α = β = 1 and γ = 0). Apparently, the morphing of EDM functions μ(*r*) possessing the same topology as single-minimum potential energy functions can be performed in just the same way. The actual morphing can be conveniently performed by fitting the “observed” matrix elements (transition moments) ⟨*vJ*|μ(*r*)|*v*′*J*′⟩, where *v* and *J* are the vibrational and rotational quantum numbers, respectively. To make this possible, a reference data set of the appropriate transition moments ⟨*vJ*|μ(*r*)|*v*′*J*′⟩_ref_ has been generated for ^12^C^16^O from the experimental data selected in ref (4), the highly accurate data given in ref (10), and the predictions for the 7-0 vibrational band obtained using the EDM function determined in ref (4), thus increasing the representativeness of this data set. Subsequently, the selected EDM functions were used to evaluate the corresponding transition moments ⟨*vJ*|μ(*r*)|*v*′*J*′⟩_calc_ and compare them with their reference counterparts (see Tables S1–S5 in the Supporting Information). The ro-vibrational wave functions were evaluated using the empirical potential energy function from Meshkov et al.,^2^ and the comparison was performed using the following quantity
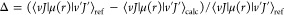
7

## 3 Results and Discussion

Strictly speaking, the EDM function μ(*r*) of CO is not a single-minimum function (see the left top panel of Figure 1 representing the “best” empirical and theoretical EDM functions from the literature), so its “formally correct” morphing using the RRC approach may appear problematical. However, when taking into account the fact that the questionable *r* → 0 asymptote cannot affect any of the bound ro-vibrational states, it becomes obvious that taking this approach is fully legitimate when morphing a dipole moment curve with respect to its local maximum at ∼2 Å.

**Figure 2:**
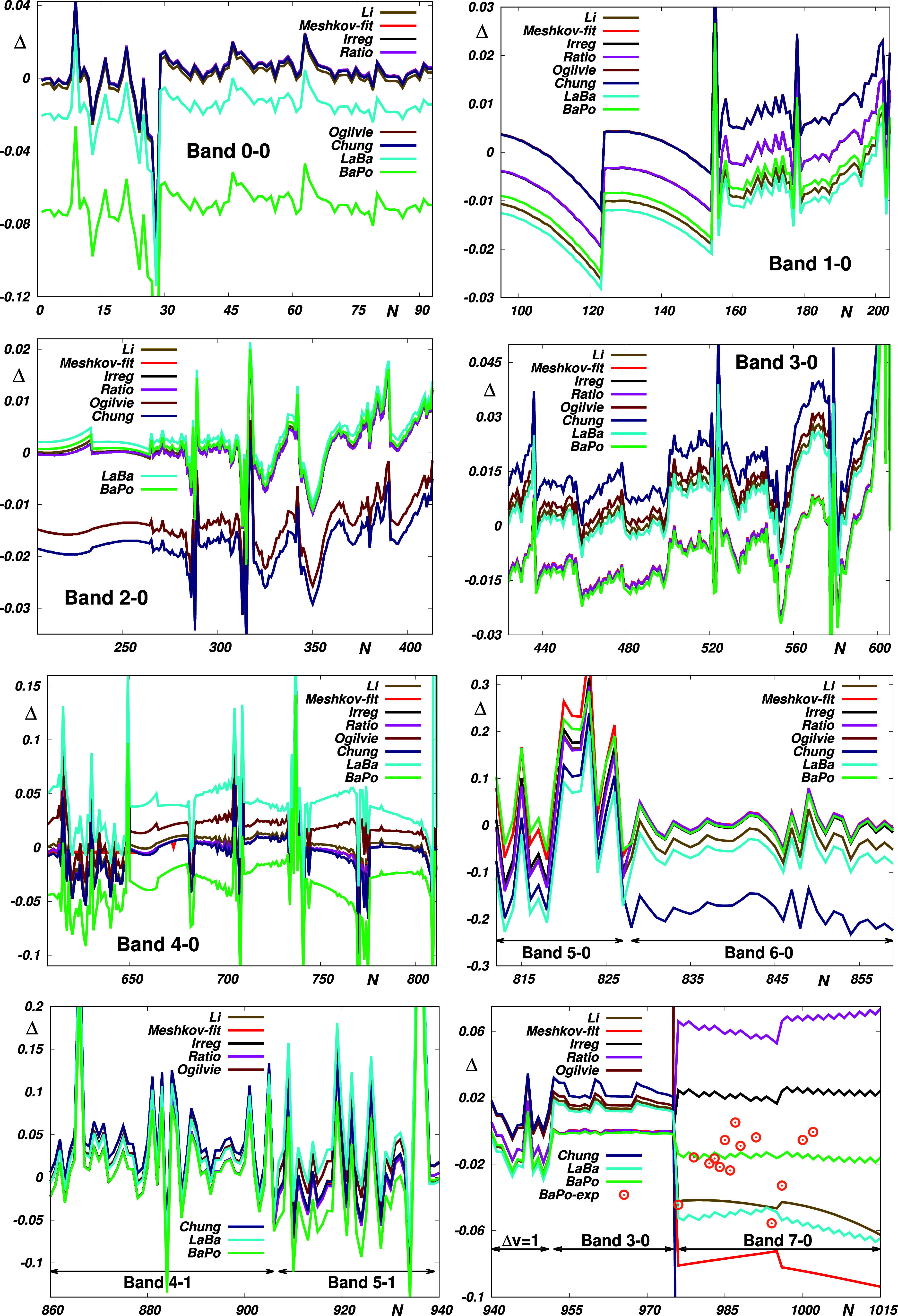
Reproduction of the CO reference data by the empirical *Li*,^1^*Meshkov-fit*,^4^*Irreg* and *Ratio*,^5^*Ogilvie*,^12^ and *Chung*(13) EDM and by their theoretical *LaBa*(14) and *BaPo*(11) counterparts.

**Figure 3:**
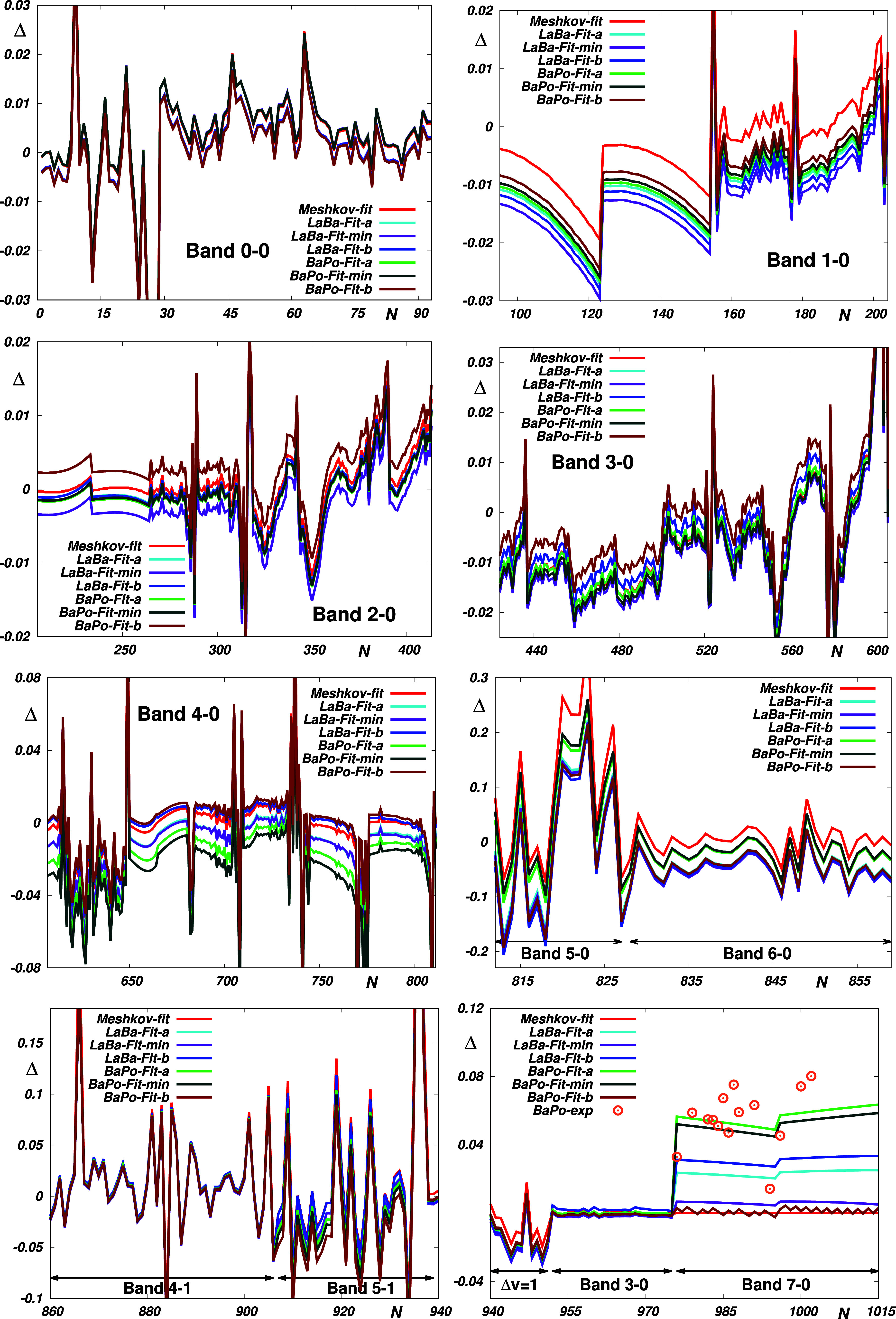
Reproduction of the CO reference data by the “best” empirical EDM (*Meshkov-fit*)^4^ and by their morphed *Fit-a*, *Fit-min*, and *Fit-b* theoretical *LaBa*(14) and *BaPo*(11) counterparts obtained by fitting the basic RRC parameters [*r*_e_, ρ_*ij*_, *D*_e_] to accurate experimental transition moments.

From the top left panel of Figure 1, it is evident that a direct visual comparison of the probed curves does not provide full insight. It appears from the top right panel of Figure 1 that better insight into their usage is provided by their deviations from the “reference” *Meshkov-fit* function.^4^ In the interval where the probed vibrational states are localized, the curves coincide within ±0.015 D. However, despite being relatively small, these deviations give rise to fairly large discrepancies between the corresponding transition moments for high overtones.^4^ As is seen from the bottom panels of Figure 1, this concerns particularly the ACPF curves in ref (4). Recently, it has been reported in ref (11) that the problem faced is caused by a “nonmonotonous behavior” of the perturbative Davidson corrections. Actually, however, as can be seen in Figure 2, the polynomial EDM function obtained in ref (11) by fitting appropriately selected ab initio points gives reliable transition moments for all spectral bands studied, similarly to all its probed semiempirical counterparts and even its “best” theoretical counterpart from ref (14). Still, however, none of these functions can describe observation data with experimental accuracy. Nevertheless, as described in ref (6), such accuracy could possibly be achieved through morphing these functions by fitting them to an experiment within the framework of the RRC approach. To keep this as “physics-guided” as possible, the actual morphing was primarily performed for ab initio curves by fitting only the basic RRC parameters [*r*_e_, ρ_*ij*_, *D*_e_] and considering only the most accurate experimental data, namely, the EDM μ̅ measured with microwave accuracy in ref (15) and the twenty-four ⟨*v* = 0, *J*|μ(*r*)|*v* = 3, *J*′⟩ transition moments derived from the intensities measured with subper-mille accuracy in ref (10) (labeled *Fit-a*). Moreover, to assess the physical robustness of the morphing performed, two additional fits of the basic RRC parameters were made: (i) a fit respecting only μ, ⟨*v* = 0, *J* = 30|μ|*v* = 3, *J* = 29⟩ and ⟨*v* = 0, *J* = 28|μ|*v* = 3, *J* = 29⟩ (labeled *Fit-min*); and (ii) a fit respecting the Δ*v* < 5 subset of all of 1015 reference data points (labeled *Fit-b*). As seen in Figure 3, using the basic RRC parameters [*r*_e_, ρ_*ij*_, *D*_e_], these fits (labeled *Fit-a*) predict the remaining reference data in excellent agreement with their best available semiempirical multiparameter counterparts. Importantly, as can be seen in Table 1, the fits performed using the best theoretical EDM (ref (11)) provide predictions for the highest (7-0) overtone with accuracy which appears to be well below the experimental uncertainty. Unfortunately, however, the uncertainties of the remaining reference data, particularly the data for some higher overtones, are much above the experimentally achievable accuracy limit. Apparently, to improve and assess the actual accuracy of the morphed functions, more accurate data are still needed for certain bands.

**Table 1:** Relative Differences (in %) between Experimental and Calculated Line Intensities in the 7-0 Band of ^12^C^16^O

line	*S*_exp_^a^	*Unct*^a^	*Li*^b^	*MeGo*^c^	*Irreg*^d^	*LaBa*^e^	*Fit-a*^f^	*Fit-b*^g^	*Fit-a*^h^	*Fit-b*^i^	*Fit-c*^j^	fit.ai0125^k^
P19	1.832	21	15.7	–2.9	14.7	2.4	1.3	2.7	7.0	7.2	8.3	10.3
P16	3.695	7.4	7.0	15.7	6.0	–7.9	–9.2	–7.6	–2.7	–2.5	–1.2	0.0
P13	6.789	4.1	8.1	–13.0	7.3	–6.8	–8.1	–6.5	–1.6	–1.4	0.0	1.2
P12	7.633	4.0	5.0	–16.9	4.2	–10.5	–11.9	–10.2	–5.0	–4.9	–3.4	–2.2
P11	9.191	4.1	10.6	–10.1	9.9	–4.0	–5.3	–3.0	1.1	1.3	2.7	4.0
P10	9.723	3.7	6.9	–14.9	6.1	–8.5	–9.9	–8.3	–3.1	–2.9	–1.5	–0.3
P9	10.80	3.3	10.1	–11.0	9.4	–4.8	–6.1	–4.5	0.5	0.6	2.1	3.4
P8	11.14	3.4	9.6	–11.9	8.9	–5.6	–6.9	–5.3	–0.1	–0.1	1.5	2.7
P7	11.18	3.4	9.6	–12.0	9.0	–5.6	–6.9	–5.3	–0.1	–0.1	1.5	2.7
P4	8.560	3.8	9.3	–12.9	8.6	–6.4	–7.7	–6.1	–0.7	–0.7	0.9	2.2
P1	2.578	11	14.4	–6.9	13.8	–0.7	–1.9	–0.3	4.8	4.8	6.4	8.2
R0	2.495	12	12.4	–9.7	11.8	–3.3	–4.6	–2.9	2.5	2.5	4.1	5.6
R4	9.243	3.5	7.5	–16.7	6.8	–9.8	–11.1	–9.3	–3.4	–3.4	–1.6	–0.2
R6	10.12	3.5	6.6	–18.2	5.8	–11.2	–12.5	–10.7	–4.6	–4.6	–2.7	–1.3

a Experimental intensities *S*_exp_ (in cm molecule^–1^) and their uncertainties *Unct* (in %) are taken from Table 3 of ref (11); all line intensities are scaled to 100% abundance of ^12^C^16^O.

b Calculated using EDM defined by Table 4 of ref (1).

c Calculated using EDM defined by Table 3 of ref (4).

d Calculated using EDM defined by Table 1 of ref (5).

e Calculated using EDM defined by Table 2 of ref (14).

f Obtained by fitting the subset of the most accurate data using EDM defined by Table 2 of ref (14).

g Obtained by fitting the subset of the Δ*v* < 5 data using EDM defined by Table 7 of ref (14).

h Obtained by fitting the subset of the most accurate data using EDM defined by Table 7 of ref (11).

i Obtained by fitting the subset of the Δ*v* < 5 data using EDM defined by Table 7 of ref (11).

j Obtained by fitting the subset of the most accurate data extended by the band 7-0 data (see Tables S4 and S5) using EDM defined by Table 7 of ref (11).

k Taken from the last column of Table 3 of ref (11).

**Figure 4:**
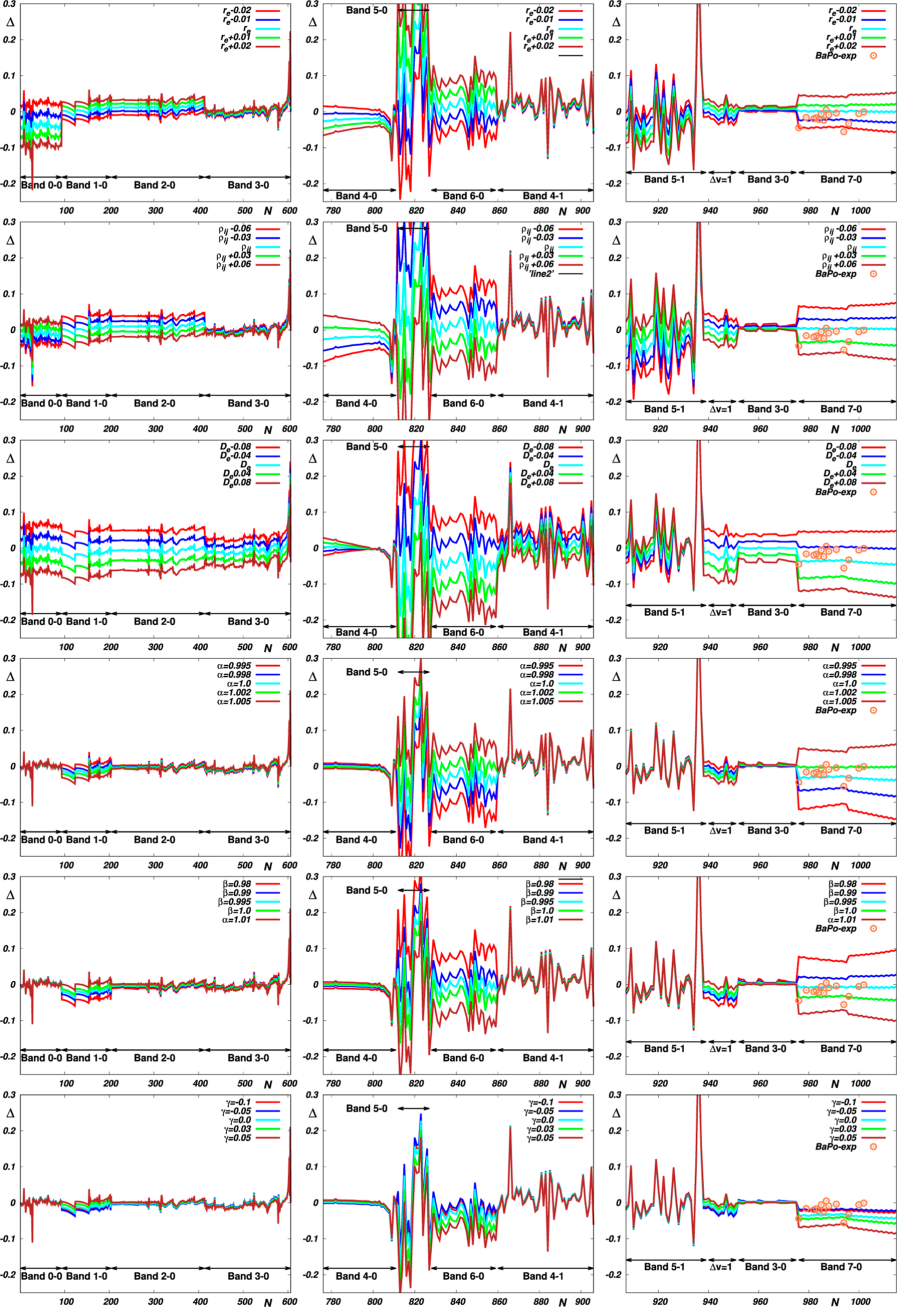
Sensitivity of the probed transition moments to variation of the fitted RRC parameters. The upper panels illustrate the results obtained by gradually fitting two basic RRC parameters with fixing the third parameter at a given value and with α = β = 1 and γ = 0; *r*_e_, ρ_*ij*_, and *D*_e_ are fixed at their ab initio values. The lower panels illustrate the results obtained by fitting all the basic parameters and varying one of the “correcting” parameters α, β, or γ.

In selecting candidates suitable for new measurements, it is worth reckoning with the fact that the intensities of different spectral transitions exhibit different sensitivities to variation in the fitted parameters of the respective dipole moment functions. To elucidate the nature of these sensitivities, additional fits were performed while the probed parameters were varied accordingly. The results of these fits, illustrated in Figure 4, show that the most sensitive spectral intensities are exhibited by high overtones, which makes their precise remeasurement highly desirable. Rather surprisingly, the results also show that the transition moments belonging to the Δ*v* = 1 bands exhibit a much higher sensitivity to changes in the correction parameters [α, β, γ] than the 2-0, 3-0, 4-0, 4-1, and 5-1 overtones. Precise measurement of the intensities of these bands thus appears to be a suitable way of assessing the role of these parameters.

## 4 Conclusions

The results described reveal two important facts:

- The RRC scheme enables the construction of physically correct EDM functions over a large range of vibrational distortions using a much smaller number of fitting parameters than the usual approach based on the use of standard elementary mathematical functions, and this applies even to functions that are complex in shape; and
- Highly accurate results can be achieved by employing standard quantum chemical methods if they correctly overcome the basis set incompleteness error.

## Supporting Information Available

Ab initio, reduced, and morphed EDM curves and reference values of ro-vibrational electric dipole matrix elements of CO (PDF)
