# Supplementary material for: Three-Parameter Electric Dipole Moment Function for the CO Molecule
Source: J Chem Theory Comput. 2024 May 22;20(11):4711–7. doi: 10.1021/acs.jctc.4c00098 (PMC11171294; doi:10.1021/acs.jctc.4c00098)
Supplement: Supplementary file 1 — ct4c00098_si_001.pdf [file ct4c00098_si_001.pdf]

Supporting Information. A three-parameter electric dipole moment function for the CO molecule

Vladimir Spirko*

Institute of Organic Chemistry and Biochemistry, p.r.i.
Czech Academy of Sciences, Flemingovo nam.~2,
166 10 Prague 6, Czechia

E-mail: spirko@uochb.cas.cz

Phone: +420 220183 571

Table S1. Smoothing the electric dipole moment function EDM of CO [S.R. Langhoff,J.C.W. Bauschlicher,J.Chem.Phys.102,5220(1995)].

r/ANG	AB INITIO	CALCULATED	AI-C	WEIGHT
0.8466836	-0.978732	-0.978918	0.000186	102.17
0.8996013	-0.833394	-0.833357	-0.000037	1199.91
0.9525190	-0.678067	-0.678055	-0.000012	1474.78
1.0054368	-0.515521	-0.515544	0.000023	1939.79
1.0583545	-0.348374	-0.348389	0.000015	2870.47
1.1112722	-0.179093	-0.179096	0.000003	5583.70
1.1377311	-0.094401	-0.094397	-0.000004	10593.09
1.1641899	-0.010040	-0.010040	-0.000000	99601.83
1.2171077	0.156598	0.156595	0.000003	6385.77
1.3229431	0.474980	0.474989	-0.000010	2105.35
1.4287786	0.763216	0.763300	-0.000084	1310.24
1.5346140	1.012436	1.012431	0.000005	987.72
1.6404495	1.216311	1.216075	0.000237	822.16
1.7462849	1.369453	1.369168	0.000285	730.22
1.8521204	1.466345	1.466453	-0.000108	681.97
1.9579558	1.501879	1.502419	-0.000540	665.83
2.0637913	1.473030	1.473400	-0.000370	678.87
2.1696267	1.381653	1.381251	0.000402	723.77
2.2754622	1.237230	1.236639	0.000591	808.26
2.3812976	1.059434	1.059841	-0.000408	943.90
2.9633926	0.628172	0.627858	0.000314	15.92

SUM OF SQUARES OF ERRORS=0.9749D-03 AND THE STANDARD DEVIATION IS=0.0087

	OLD PARM	NEW PARM	DELTA PARM	ST. ERR.
r_e	0.196402642D+01	0.196402642D+01	0.30465D-12	0.17540D-03
C[2]	-0.225936527D+01	-0.225936527D+01	-0.11197D-10	0.16866D-02
C[3]	-0.111198138D+01	-0.111198138D+01	-0.30557D-09	0.25291D-01
C[5]	0.154453173D+01	0.154453173D+01	0.30000D-09	0.24732D-01
C[6]	0.176959839D+01	0.176959839D+01	0.40444D-09	0.31527D-01
C[7]	0.907679887D+00	0.907679888D+00	0.18532D-09	0.84834D-02
a2	0.372235227D+00	0.372235227D+00	-0.81531D-10	0.58438D-02
V0	0.150252653D+01	0.150252653D+01	0.33840D-12	0.17491D-03

(C[4]=0.0 C[8]=0.190 a=1.1380, fixed after a preliminary determination)

$$y=1-exp\{a*(r-r_e)+a2*(r-r_e)^2$$

$$EDM=V0+C[2]*y^2+C[3]*y^3+C[4]*y^4+C[5]*y^5+C[6]*y^6+C[7]*y^7+C[8]*y^8$$

Table S2. The parameters of the morphed electric dipole moment functions of CO
[S.R. Langhoff,J.C.W. Bauschlicher,J.Chem.Phys.102,5220(1995)].

Parameter	Fit-0(a)	Fit-a	Fit-b	Fit-c	Fit-d	Fit-d
r_e, Å	1.963824	1.97098(78)	1.96942(30)	1.96815(28)	1.97448(29)	1.97381(29)
ρ_{ij} (κ=-3), Å	1.465919	1.4456(14)	1.44946(52)	1.43775(47)	1.41364(48)	1.39851(46)
D_e, D	1.502003	1.50634(81)	1.50622(65)	1.48068(60)	1.48750(61)	1.50187(63)
α	1.0	1.0	1.0	0.995(b)	1.0	1.0
β	1.0	1.0	1.0	1.0	1.010(b)	1.0
γ	0.0	0.0	0.0	0.0	0.0	0.014(b)
σ(c), D	0.03311	0.01640	0.01548	0.01486	0.01487	0.01512

(a) Ab Initio values. (b) Fixed after a preliminary determination.
(b) The standard deviation of the fit.

Table S3. The reproduction of the reference transition dipole moments (in Debye) TDM
of the $X^1Σ^+$ state of 12C16O by the Fit-b (see Table S2) electric dipole moment function
of S.R. Langhoff,J.C.W. Bauschlicher,J.Chem.Phys.102,5220(1995) (see Tables S1 and S2).

Trans.	v"	J"	v`	J`	TDM-ref/D	TDM-calc/D	ref-calc/D	Weight	Δ[a]
1	0	0	0	0	-0.1097000D+00	-0.1099899D+00	0.2899026D-03	0.83D+02	-0.002643
2	0	0	0	0	-0.1098000D+00	-0.1099899D+00	0.1899026D-03	0.83D+02	-0.001730
3	0	0	0	0	-0.1098200D+00	-0.1099899D+00	0.1699026D-03	0.83D+02	-0.001547
4	0	0	0	0	-0.1094500D+00	-0.1099899D+00	0.5399026D-03	0.83D+02	-0.004933
5	0	0	0	0	-0.1097600D+00	-0.1099899D+00	0.2299026D-03	0.83D+02	-0.002095
6	0	0	0	0	-0.1095400D+00	-0.1099899D+00	0.4499026D-03	0.83D+02	-0.004107
7	0	0	0	0	-0.1095100D+00	-0.1099899D+00	0.4799026D-03	0.83D+02	-0.004382
8	0	0	0	0	-0.1101300D+00	-0.1099899D+00	-0.1400974D-03	0.82D+02	0.001272
9	0	2	0	3	-0.1146129D+00	-0.1098864D+00	-0.4726508D-02	0.76D+02	0.041239
10	0	3	0	4	-0.1096788D+00	-0.1098059D+00	0.1270803D-03	0.83D+02	-0.001159
11	0	4	0	5	-0.1101668D+00	-0.1097024D+00	-0.4644403D-03	0.82D+02	0.004216
12	0	5	0	6	-0.1094524D+00	-0.1095758D+00	0.1234268D-03	0.83D+02	-0.001128
13	0	6	0	7	-0.1067608D+00	-0.1094263D+00	0.2665477D-02	0.88D+02	-0.024967
14	0	7	0	8	-0.1081163D+00	-0.1092537D+00	0.1137404D-02	0.86D+02	-0.010520
15	0	8	0	9	-0.1088592D+00	-0.1090581D+00	0.1989022D-03	0.84D+02	-0.001827
16	0	9	0	10	-0.1098914D+00	-0.1088395D+00	-0.1051936D-02	0.83D+02	0.009573
17	0	10	0	11	-0.1081358D+00	-0.1085978D+00	0.4619817D-03	0.86D+02	-0.004272
18	0	11	0	12	-0.1078610D+00	-0.1083330D+00	0.4720457D-03	0.86D+02	-0.004376
19	0	12	0	13	-0.1074163D+00	-0.1080452D+00	0.6289465D-03	0.87D+02	-0.005855
20	0	13	0	14	-0.1079059D+00	-0.1077344D+00	-0.1715266D-03	0.86D+02	0.001590
21	0	14	0	15	-0.1091445D+00	-0.1074004D+00	-0.1744085D-02	0.84D+02	0.015980
22	0	15	0	16	-0.1068145D+00	-0.1070434D+00	0.2288581D-03	0.88D+02	-0.002143
23	0	16	0	17	-0.1053297D+00	-0.1066632D+00	0.1333490D-02	0.90D+02	-0.012660
24	0	17	0	18	-0.1031894D+00	-0.1062599D+00	0.3070497D-02	0.94D+02	-0.029756
25	0	18	0	19	-0.1056987D+00	-0.1058335D+00	0.1347633D-03	0.90D+02	-0.001275
26	0	19	0	20	-0.1020549D+00	-0.1053839D+00	0.3328973D-02	0.96D+02	-0.032619
27	0	20	0	21	-0.1013656D+00	-0.1049111D+00	0.3545510D-02	0.97D+02	-0.034977
28	0	21	0	22	-0.9429411D-01	-0.1044152D+00	0.1012105D-01	0.11D+03	-0.107335
29	0	7	0	8	-0.1105115D+00	-0.1092537D+00	-0.1257796D-02	0.82D+02	0.011382
30	0	8	0	9	-0.1104989D+00	-0.1090581D+00	-0.1440798D-02	0.82D+02	0.013039
31	0	9	0	10	-0.1097878D+00	-0.1088395D+00	-0.9483358D-03	0.83D+02	0.008638
32	0	10	0	11	-0.1093017D+00	-0.1085978D+00	-0.7039183D-03	0.84D+02	0.006440
33	0	11	0	12	-0.1092147D+00	-0.1083330D+00	-0.8816543D-03	0.84D+02	0.008073
34	0	12	0	13	-0.1085564D+00	-0.1080452D+00	-0.5111535D-03	0.85D+02	0.004709
35	0	13	0	14	-0.1080114D+00	-0.1077344D+00	-0.2770266D-03	0.86D+02	0.002565
36	0	14	0	15	-0.1074134D+00	-0.1074004D+00	-0.1298529D-04	0.87D+02	0.000121
37	0	15	0	16	-0.1076772D+00	-0.1070434D+00	-0.6338419D-03	0.86D+02	0.005887
38	0	16	0	17	-0.1069408D+00	-0.1066632D+00	-0.2776098D-03	0.87D+02	0.002596
39	0	17	0	18	-0.1061207D+00	-0.1062599D+00	0.1391970D-03	0.89D+02	-0.001312
40	0	18	0	19	-0.1060684D+00	-0.1058335D+00	-0.2349367D-03	0.89D+02	0.002215
41	0	19	0	20	-0.1056871D+00	-0.1053839D+00	-0.3032266D-03	0.90D+02	0.002869
42	0	20	0	21	-0.1055485D+00	-0.1049111D+00	-0.6373897D-03	0.90D+02	0.006039
43	0	21	0	22	-0.1044277D+00	-0.1044152D+00	-0.1254343D-04	0.92D+02	0.000120
44	0	22	0	23	-0.1045104D+00	-0.1038960D+00	-0.6144064D-03	0.92D+02	0.005879

45	0	23	0	24	-0.1040956D+00	-0.1033536D+00	-0.7419980D-03	0.92D+02	0.007128
46	0	7	0	8	-0.1112841D+00	-0.1092537D+00	-0.2030396D-02	0.81D+02	0.018245
47	0	8	0	9	-0.1103560D+00	-0.1090581D+00	-0.1297898D-02	0.82D+02	0.011761
48	0	9	0	10	-0.1102486D+00	-0.1088395D+00	-0.1409136D-02	0.82D+02	0.012781
49	0	10	0	11	-0.1097205D+00	-0.1085978D+00	-0.1122718D-02	0.83D+02	0.010233
50	0	11	0	12	-0.1091554D+00	-0.1083330D+00	-0.8223543D-03	0.84D+02	0.007534
51	0	12	0	13	-0.1084968D+00	-0.1080452D+00	-0.4515535D-03	0.85D+02	0.004162
52	0	13	0	14	-0.1084776D+00	-0.1077344D+00	-0.7432266D-03	0.85D+02	0.006851
53	0	14	0	15	-0.1081527D+00	-0.1074004D+00	-0.7522853D-03	0.85D+02	0.006956
54	0	15	0	16	-0.1076088D+00	-0.1070434D+00	-0.5654419D-03	0.86D+02	0.005255
55	0	16	0	17	-0.1074668D+00	-0.1066632D+00	-0.8036098D-03	0.87D+02	0.007478
56	0	17	0	18	-0.1062943D+00	-0.1062599D+00	-0.3440301D-04	0.89D+02	0.000324
57	0	18	0	19	-0.1066800D+00	-0.1058335D+00	-0.8465367D-03	0.88D+02	0.007935
58	0	19	0	20	-0.1062250D+00	-0.1053839D+00	-0.8411266D-03	0.89D+02	0.007918
59	0	20	0	21	-0.1060195D+00	-0.1049111D+00	-0.1108390D-02	0.89D+02	0.010455
60	0	21	0	22	-0.1051612D+00	-0.1044152D+00	-0.7460434D-03	0.90D+02	0.007094
61	0	22	0	23	-0.1039486D+00	-0.1038960D+00	-0.5260639D-04	0.93D+02	0.000506
62	0	23	0	24	-0.1036965D+00	-0.1033536D+00	-0.3428980D-03	0.93D+02	0.003307
63	0	7	0	8	-0.1117800D+00	-0.1092537D+00	-0.2526296D-02	0.80D+02	0.022601
64	0	8	0	9	-0.1106570D+00	-0.1090581D+00	-0.1598898D-02	0.82D+02	0.014449
65	0	9	0	10	-0.1099689D+00	-0.1088395D+00	-0.1129436D-02	0.83D+02	0.010271
66	0	10	0	11	-0.1092588D+00	-0.1085978D+00	-0.6610183D-03	0.84D+02	0.006050
67	0	11	0	12	-0.1088741D+00	-0.1083330D+00	-0.5410543D-03	0.84D+02	0.004970
68	0	12	0	13	-0.1085342D+00	-0.1080452D+00	-0.4889535D-03	0.85D+02	0.004505
69	0	13	0	14	-0.1080915D+00	-0.1077344D+00	-0.3571266D-03	0.86D+02	0.003304
70	0	14	0	15	-0.1072864D+00	-0.1074004D+00	0.1140147D-03	0.87D+02	-0.001063
71	0	15	0	16	-0.1073425D+00	-0.1070434D+00	-0.2991419D-03	0.87D+02	0.002787
72	0	16	0	17	-0.1069944D+00	-0.1066632D+00	-0.3312098D-03	0.87D+02	0.003096
73	0	17	0	18	-0.1066013D+00	-0.1062599D+00	-0.3414030D-03	0.88D+02	0.003203
74	0	18	0	19	-0.1057536D+00	-0.1058335D+00	0.7986332D-04	0.89D+02	-0.000755
75	0	19	0	20	-0.1058672D+00	-0.1053839D+00	-0.4833266D-03	0.89D+02	0.004565
76	0	20	0	21	-0.1050105D+00	-0.1049111D+00	-0.9938968D-04	0.91D+02	0.000946
77	0	21	0	22	-0.1043932D+00	-0.1044152D+00	0.2195657D-04	0.92D+02	-0.000210
78	0	22	0	23	-0.1040578D+00	-0.1038960D+00	-0.1618064D-03	0.92D+02	0.001555
79	0	23	0	24	-0.1028163D+00	-0.1033536D+00	0.5373020D-03	0.95D+02	-0.005226
80	0	8	0	9	-0.1098535D+00	-0.1090581D+00	-0.7953978D-03	0.83D+02	0.007241
81	0	9	0	10	-0.1092326D+00	-0.1088395D+00	-0.3931358D-03	0.84D+02	0.003599
82	0	10	0	11	-0.1085956D+00	-0.1085978D+00	0.2181659D-05	0.85D+02	-0.000020
83	0	11	0	12	-0.1082879D+00	-0.1083330D+00	0.4514573D-04	0.85D+02	-0.000417
84	0	12	0	13	-0.1080307D+00	-0.1080452D+00	0.1454654D-04	0.86D+02	-0.000135
85	0	13	0	14	-0.1076773D+00	-0.1077344D+00	0.5707338D-04	0.86D+02	-0.000530
86	0	14	0	15	-0.1069682D+00	-0.1074004D+00	0.4322147D-03	0.87D+02	-0.004041
87	0	15	0	16	-0.1071236D+00	-0.1070434D+00	-0.8024192D-04	0.87D+02	0.000749
88	0	16	0	17	-0.1068818D+00	-0.1066632D+00	-0.2186098D-03	0.88D+02	0.002045
89	0	17	0	18	-0.1066008D+00	-0.1062599D+00	-0.3409030D-03	0.88D+02	0.003198
90	0	18	0	19	-0.1058703D+00	-0.1058335D+00	-0.3683668D-04	0.89D+02	0.000348
91	0	19	0	20	-0.1061079D+00	-0.1053839D+00	-0.7240266D-03	0.89D+02	0.006823
92	0	20	0	21	-0.1053785D+00	-0.1049111D+00	-0.4673897D-03	0.90D+02	0.004435
93	0	21	0	22	-0.1048939D+00	-0.1044152D+00	-0.4787434D-03	0.91D+02	0.004564
94	0	1	1	0	-0.1063450D+00	-0.1075655D+00	0.1220517D-02	0.88D+02	-0.011477
95	0	2	1	1	-0.1063100D+00	-0.1075563D+00	0.1246310D-02	0.88D+02	-0.011723
96	0	3	1	2	-0.1062820D+00	-0.1075479D+00	0.1265872D-02	0.89D+02	-0.011910
97	0	4	1	3	-0.1062600D+00	-0.1075402D+00	0.1280207D-02	0.89D+02	-0.012048
98	0	5	1	4	-0.1062300D+00	-0.1075333D+00	0.1303325D-02	0.89D+02	-0.012269
99	0	6	1	5	-0.1061950D+00	-0.1075272D+00	0.1332231D-02	0.89D+02	-0.012545
100	0	7	1	6	-0.1061500D+00	-0.1075219D+00	0.1371933D-02	0.89D+02	-0.012924
101	0	8	1	7	-0.1061210D+00	-0.1075174D+00	0.1396438D-02	0.89D+02	-0.013159
102	0	9	1	8	-0.1060830D+00	-0.1075138D+00	0.1430754D-02	0.89D+02	-0.013487
103	0	10	1	9	-0.1060290D+00	-0.1075109D+00	0.1481886D-02	0.89D+02	-0.013976
104	0	11	1	10	-0.1059810D+00	-0.1075088D+00	0.1527843D-02	0.89D+02	-0.014416
105	0	12	1	11	-0.1059390D+00	-0.1075076D+00	0.1568631D-02	0.89D+02	-0.014807
106	0	13	1	12	-0.1058970D+00	-0.1075073D+00	0.1610257D-02	0.89D+02	-0.015206
107	0	14	1	13	-0.1058400D+00	-0.1075077D+00	0.1667729D-02	0.89D+02	-0.015757
108	0	15	1	14	-0.1058040D+00	-0.1075091D+00	0.1705052D-02	0.89D+02	-0.016115
109	0	16	1	15	-0.1057520D+00	-0.1075112D+00	0.1759235D-02	0.89D+02	-0.016635
110	0	17	1	16	-0.1056720D+00	-0.1075143D+00	0.1842284D-02	0.90D+02	-0.017434
111	0	18	1	17	-0.1056140D+00	-0.1075182D+00	0.1904205D-02	0.90D+02	-0.018030
112	0	19	1	18	-0.1055430D+00	-0.1075230D+00	0.1980007D-02	0.90D+02	-0.018760
113	0	20	1	19	-0.1054830D+00	-0.1075287D+00	0.2045695D-02	0.90D+02	-0.019394
114	0	21	1	20	-0.1054080D+00	-0.1075353D+00	0.2127277D-02	0.90D+02	-0.020181
115	0	22	1	21	-0.1053510D+00	-0.1075428D+00	0.2191760D-02	0.90D+02	-0.020804

116	0	23	1	22	-0.1052520D+00	-0.1075511D+00	0.2299150D-02	0.90D+02	-0.021844
117	0	24	1	23	-0.1052050D+00	-0.1075605D+00	0.2355454D-02	0.90D+02	-0.022389
118	0	25	1	24	-0.1051250D+00	-0.1075707D+00	0.2445679D-02	0.90D+02	-0.023264
119	0	26	1	25	-0.1050510D+00	-0.1075818D+00	0.2530832D-02	0.91D+02	-0.024091
120	0	27	1	26	-0.1049560D+00	-0.1075939D+00	0.2637919D-02	0.91D+02	-0.025134
121	0	28	1	27	-0.1049020D+00	-0.1076069D+00	0.2704949D-02	0.91D+02	-0.025785
122	0	29	1	28	-0.1047990D+00	-0.1076209D+00	0.2821926D-02	0.91D+02	-0.026927
123	0	30	1	29	-0.1047100D+00	-0.1076359D+00	0.2925859D-02	0.91D+02	-0.027942
124	0	0	1	1	-0.1063880D+00	-0.1075862D+00	0.1198202D-02	0.88D+02	-0.011263
125	0	1	1	2	-0.1064170D+00	-0.1075977D+00	0.1180667D-02	0.88D+02	-0.011095
126	0	2	1	3	-0.1064220D+00	-0.1076099D+00	0.1187870D-02	0.88D+02	-0.011162
127	0	3	1	4	-0.1064390D+00	-0.1076228D+00	0.1183804D-02	0.88D+02	-0.011122
128	0	4	1	5	-0.1064530D+00	-0.1076365D+00	0.1183462D-02	0.88D+02	-0.011117
129	0	5	1	6	-0.1064750D+00	-0.1076508D+00	0.1175837D-02	0.88D+02	-0.011043
130	0	6	1	7	-0.1064820D+00	-0.1076659D+00	0.1183920D-02	0.88D+02	-0.011118
131	0	7	1	8	-0.1064900D+00	-0.1076817D+00	0.1191704D-02	0.88D+02	-0.011191
132	0	8	1	9	-0.1064840D+00	-0.1076982D+00	0.1214183D-02	0.88D+02	-0.011402
133	0	9	1	10	-0.1064910D+00	-0.1077153D+00	0.1224348D-02	0.88D+02	-0.011497
134	0	10	1	11	-0.1065010D+00	-0.1077332D+00	0.1232192D-02	0.88D+02	-0.011570
135	0	11	1	12	-0.1064910D+00	-0.1077517D+00	0.1260707D-02	0.88D+02	-0.011839
136	0	12	1	13	-0.1064960D+00	-0.1077709D+00	0.1274886D-02	0.88D+02	-0.011971
137	0	13	1	14	-0.1064820D+00	-0.1077907D+00	0.1308722D-02	0.88D+02	-0.012291
138	0	14	1	15	-0.1064760D+00	-0.1078112D+00	0.1335205D-02	0.88D+02	-0.012540
139	0	15	1	16	-0.1064650D+00	-0.1078323D+00	0.1367329D-02	0.88D+02	-0.012843
140	0	16	1	17	-0.1064620D+00	-0.1078541D+00	0.1392087D-02	0.88D+02	-0.013076
141	0	17	1	18	-0.1064430D+00	-0.1078765D+00	0.1433469D-02	0.88D+02	-0.013467
142	0	18	1	19	-0.1064250D+00	-0.1078995D+00	0.1474469D-02	0.88D+02	-0.013855
143	0	19	1	20	-0.1064080D+00	-0.1079231D+00	0.1515078D-02	0.88D+02	-0.014238
144	0	20	1	21	-0.1063890D+00	-0.1079473D+00	0.1558288D-02	0.88D+02	-0.014647
145	0	21	1	22	-0.1063700D+00	-0.1079721D+00	0.1602092D-02	0.88D+02	-0.015062
146	0	22	1	23	-0.1063420D+00	-0.1079975D+00	0.1655482D-02	0.88D+02	-0.015568
147	0	23	1	24	-0.1063270D+00	-0.1080234D+00	0.1696448D-02	0.88D+02	-0.015955
148	0	24	1	25	-0.1062800D+00	-0.1080500D+00	0.1769984D-02	0.89D+02	-0.016654
149	0	25	1	26	-0.1062500D+00	-0.1080771D+00	0.1827082D-02	0.89D+02	-0.017196
150	0	26	1	27	-0.1062230D+00	-0.1081047D+00	0.1881732D-02	0.89D+02	-0.017715
151	0	27	1	28	-0.1061750D+00	-0.1081329D+00	0.1957926D-02	0.89D+02	-0.018441
152	0	28	1	29	-0.1061510D+00	-0.1081617D+00	0.2010657D-02	0.89D+02	-0.018941
153	0	29	1	30	-0.1061040D+00	-0.1081909D+00	0.2086916D-02	0.89D+02	-0.019669
154	0	30	1	31	-0.1060670D+00	-0.1082207D+00	0.2153694D-02	0.89D+02	-0.020305
155	0	4	1	3	-0.1101810D+00	-0.1075402D+00	-0.2640793D-02	0.82D+02	0.023968
156	0	1	1	0	-0.1058134D+00	-0.1075655D+00	0.1752117D-02	0.89D+02	-0.016559
157	0	2	1	1	-0.1067340D+00	-0.1075563D+00	0.8223103D-03	0.88D+02	-0.007704
158	0	3	1	2	-0.1070799D+00	-0.1075479D+00	0.4679715D-03	0.87D+02	-0.004370
159	0	4	1	3	-0.1065032D+00	-0.1075402D+00	0.1037007D-02	0.88D+02	-0.009737
160	0	5	1	4	-0.1064427D+00	-0.1075333D+00	0.1090625D-02	0.88D+02	-0.010246
161	0	6	1	5	-0.1064436D+00	-0.1075272D+00	0.1083631D-02	0.88D+02	-0.010180
162	0	7	1	6	-0.1064196D+00	-0.1075219D+00	0.1102333D-02	0.88D+02	-0.010358
163	0	8	1	7	-0.1063936D+00	-0.1075174D+00	0.1123838D-02	0.88D+02	-0.010563
164	0	9	1	8	-0.1065948D+00	-0.1075138D+00	0.9189537D-03	0.88D+02	-0.008621
165	0	10	1	9	-0.1064840D+00	-0.1075109D+00	0.1026886D-02	0.88D+02	-0.009644
166	0	11	1	10	-0.1066752D+00	-0.1075088D+00	0.8336430D-03	0.88D+02	-0.007815
167	0	12	1	11	-0.1068630D+00	-0.1075076D+00	0.6446310D-03	0.88D+02	-0.006032
168	0	13	1	12	-0.1066509D+00	-0.1075073D+00	0.8563573D-03	0.88D+02	-0.008030
169	0	14	1	13	-0.1069245D+00	-0.1075077D+00	0.5832287D-03	0.87D+02	-0.005455
170	0	15	1	14	-0.1067497D+00	-0.1075091D+00	0.7593523D-03	0.88D+02	-0.007113
171	0	16	1	15	-0.1066714D+00	-0.1075112D+00	0.8398350D-03	0.88D+02	-0.007873
172	0	17	1	16	-0.1070737D+00	-0.1075143D+00	0.4405837D-03	0.87D+02	-0.004115
173	0	18	1	17	-0.1067340D+00	-0.1075182D+00	0.7842054D-03	0.88D+02	-0.007347
174	0	19	1	18	-0.1071408D+00	-0.1075230D+00	0.3822070D-03	0.87D+02	-0.003567
175	0	20	1	19	-0.1067415D+00	-0.1075287D+00	0.7871953D-03	0.88D+02	-0.007375
176	0	21	1	20	-0.1067726D+00	-0.1075353D+00	0.7626773D-03	0.88D+02	-0.007143
177	0	22	1	21	-0.1059973D+00	-0.1075428D+00	0.1545460D-02	0.89D+02	-0.014580
178	0	23	1	22	-0.1084794D+00	-0.1075511D+00	-0.9282503D-03	0.85D+02	0.008557
179	0	0	1	1	-0.1063290D+00	-0.1075862D+00	0.1257202D-02	0.88D+02	-0.011824
180	0	1	1	2	-0.1065581D+00	-0.1075977D+00	0.1039567D-02	0.88D+02	-0.009756
181	0	2	1	3	-0.1065983D+00	-0.1076099D+00	0.1011570D-02	0.88D+02	-0.009490
182	0	3	1	4	-0.1066463D+00	-0.1076228D+00	0.9765044D-03	0.88D+02	-0.009156
183	0	4	1	5	-0.1067327D+00	-0.1076365D+00	0.9037624D-03	0.88D+02	-0.008468
184	0	5	1	6	-0.1066576D+00	-0.1076508D+00	0.9932366D-03	0.88D+02	-0.009312
185	0	6	1	7	-0.1067846D+00	-0.1076659D+00	0.8813197D-03	0.88D+02	-0.008253
186	0	7	1	8	-0.1067740D+00	-0.1076817D+00	0.9077043D-03	0.88D+02	-0.008501

187	0	8	1	9	-0.1066854D+00	-0.1076982D+00	0.1012783D-02	0.88D+02	-0.009493
188	0	9	1	10	-0.1069097D+00	-0.1077153D+00	0.8056479D-03	0.87D+02	-0.007536
189	0	10	1	11	-0.1069500D+00	-0.1077332D+00	0.7831919D-03	0.87D+02	-0.007323
190	0	11	1	12	-0.1071545D+00	-0.1077517D+00	0.5972073D-03	0.87D+02	-0.005573
191	0	12	1	13	-0.1071845D+00	-0.1077709D+00	0.5863864D-03	0.87D+02	-0.005471
192	0	13	1	14	-0.1072713D+00	-0.1077907D+00	0.5194216D-03	0.87D+02	-0.004842
193	0	14	1	15	-0.1073879D+00	-0.1078112D+00	0.4233052D-03	0.87D+02	-0.003942
194	0	15	1	16	-0.1074793D+00	-0.1078323D+00	0.3530295D-03	0.87D+02	-0.003285
195	0	16	1	17	-0.1074274D+00	-0.1078541D+00	0.4266867D-03	0.87D+02	-0.003972
196	0	17	1	18	-0.1072138D+00	-0.1078765D+00	0.6626691D-03	0.87D+02	-0.006181
197	0	18	1	19	-0.1077569D+00	-0.1078995D+00	0.1425687D-03	0.86D+02	-0.001323
198	0	19	1	20	-0.1077763D+00	-0.1079231D+00	0.1467777D-03	0.86D+02	-0.001362
199	0	20	1	21	-0.1080022D+00	-0.1079473D+00	-0.5491179D-04	0.86D+02	0.000508
200	0	21	1	22	-0.1080048D+00	-0.1079721D+00	-0.3270783D-04	0.86D+02	0.000303
201	0	22	1	23	-0.1086680D+00	-0.1079975D+00	-0.6705184D-03	0.85D+02	0.006170
202	0	23	1	24	-0.1088071D+00	-0.1080234D+00	-0.7836516D-03	0.84D+02	0.007202
203	0	24	1	25	-0.1067745D+00	-0.1080500D+00	0.1275484D-02	0.88D+02	-0.011946
204	0	25	1	26	-0.1086010D+00	-0.1080771D+00	-0.5239185D-03	0.85D+02	0.004824
205	0	1	2	0	-0.6527614D-02	-0.6534672D-02	0.7058142D-05	0.23D+05	-0.001081
206	0	2	2	1	-0.6511034D-02	-0.6518166D-02	0.7131918D-05	0.24D+05	-0.001095
207	0	3	2	2	-0.6494714D-02	-0.6501843D-02	0.7128733D-05	0.24D+05	-0.001098
208	0	4	2	3	-0.6478430D-02	-0.6485702D-02	0.7272442D-05	0.24D+05	-0.001123
209	0	5	2	4	-0.6462485D-02	-0.6469745D-02	0.7259943D-05	0.24D+05	-0.001123
210	0	6	2	5	-0.6446684D-02	-0.6453970D-02	0.7286118D-05	0.24D+05	-0.001130
211	0	7	2	6	-0.6431111D-02	-0.6438378D-02	0.7266859D-05	0.24D+05	-0.001130
212	0	8	2	7	-0.6415830D-02	-0.6422968D-02	0.7138104D-05	0.24D+05	-0.001113
213	0	9	2	8	-0.6400655D-02	-0.6407741D-02	0.7085761D-05	0.24D+05	-0.001107
214	0	10	2	9	-0.6385708D-02	-0.6392696D-02	0.6987779D-05	0.25D+05	-0.001094
215	0	11	2	10	-0.6371062D-02	-0.6377833D-02	0.6771105D-05	0.25D+05	-0.001063
216	0	12	2	11	-0.6356663D-02	-0.6363153D-02	0.6489708D-05	0.25D+05	-0.001021
217	0	13	2	12	-0.6342500D-02	-0.6348655D-02	0.6154556D-05	0.25D+05	-0.000970
218	0	14	2	13	-0.6328467D-02	-0.6334339D-02	0.5871629D-05	0.25D+05	-0.000928
219	0	15	2	14	-0.6314758D-02	-0.6320205D-02	0.5446937D-05	0.25D+05	-0.000863
220	0	16	2	15	-0.6301268D-02	-0.6306253D-02	0.4985487D-05	0.25D+05	-0.000791
221	0	17	2	16	-0.6288010D-02	-0.6292484D-02	0.4474302D-05	0.25D+05	-0.000712
222	0	18	2	17	-0.6275041D-02	-0.6278897D-02	0.3856413D-05	0.25D+05	-0.000615
223	0	19	2	18	-0.6262344D-02	-0.6265493D-02	0.3148857D-05	0.25D+05	-0.000503
224	0	20	2	19	-0.6249919D-02	-0.6252271D-02	0.2351714D-05	0.26D+05	-0.000376
225	0	21	2	20	-0.6237652D-02	-0.6239231D-02	0.1579027D-05	0.26D+05	-0.000253
226	0	22	2	21	-0.6225696D-02	-0.6226374D-02	0.6778958D-06	0.26D+05	-0.000109
227	0	23	2	22	-0.6214042D-02	-0.6213699D-02	-0.3425874D-06	0.26D+05	0.000055
228	0	24	2	23	-0.6202626D-02	-0.6201208D-02	-0.1418312D-05	0.26D+05	0.000229
229	0	25	2	24	-0.6191538D-02	-0.6188899D-02	-0.2639166D-05	0.26D+05	0.000426
230	0	26	2	25	-0.6180697D-02	-0.6176773D-02	-0.3924025D-05	0.26D+05	0.000635
231	0	27	2	26	-0.6170163D-02	-0.6164830D-02	-0.5332732D-05	0.26D+05	0.000864
232	0	28	2	27	-0.6159820D-02	-0.6153071D-02	-0.6749132D-05	0.26D+05	0.001096
233	0	29	2	28	-0.6149807D-02	-0.6141495D-02	-0.8312060D-05	0.26D+05	0.001352
234	0	0	2	1	-0.6561419D-02	-0.6568234D-02	0.6815262D-05	0.23D+05	-0.001039
235	0	1	2	2	-0.6578600D-02	-0.6585290D-02	0.6690490D-05	0.23D+05	-0.001017
236	0	2	2	3	-0.6595998D-02	-0.6602530D-02	0.6532386D-05	0.23D+05	-0.000990
237	0	3	2	4	-0.6613522D-02	-0.6619954D-02	0.6432150D-05	0.23D+05	-0.000973
238	0	4	2	5	-0.6631272D-02	-0.6637562D-02	0.6289978D-05	0.23D+05	-0.000949
239	0	5	2	6	-0.6649142D-02	-0.6655354D-02	0.6212094D-05	0.23D+05	-0.000934
240	0	6	2	7	-0.6667256D-02	-0.6673331D-02	0.6074718D-05	0.22D+05	-0.000911
241	0	7	2	8	-0.6685467D-02	-0.6691492D-02	0.6025075D-05	0.22D+05	-0.000901
242	0	8	2	9	-0.6703984D-02	-0.6709838D-02	0.5854423D-05	0.22D+05	-0.000873
243	0	9	2	10	-0.6722578D-02	-0.6728370D-02	0.5792016D-05	0.22D+05	-0.000862
244	0	10	2	11	-0.6741263D-02	-0.6747087D-02	0.5824139D-05	0.22D+05	-0.000864
245	0	11	2	12	-0.6760208D-02	-0.6765990D-02	0.5782063D-05	0.22D+05	-0.000855
246	0	12	2	13	-0.6779314D-02	-0.6785079D-02	0.5765094D-05	0.22D+05	-0.000850
247	0	13	2	14	-0.6798415D-02	-0.6804355D-02	0.5939529D-05	0.22D+05	-0.000874
248	0	14	2	15	-0.6817908D-02	-0.6823817D-02	0.5908688D-05	0.22D+05	-0.000867
249	0	15	2	16	-0.6837386D-02	-0.6843466D-02	0.6079905D-05	0.21D+05	-0.000889
250	0	16	2	17	-0.6857187D-02	-0.6863303D-02	0.6115528D-05	0.21D+05	-0.000892
251	0	17	2	18	-0.6876865D-02	-0.6883327D-02	0.6461910D-05	0.21D+05	-0.000940
252	0	18	2	19	-0.6896848D-02	-0.6903539D-02	0.6691410D-05	0.21D+05	-0.000970
253	0	19	2	20	-0.6916939D-02	-0.6923940D-02	0.7001399D-05	0.21D+05	-0.001012
254	0	20	2	21	-0.6937117D-02	-0.6944530D-02	0.7413296D-05	0.21D+05	-0.001069
255	0	21	2	22	-0.6957438D-02	-0.6965309D-02	0.7871471D-05	0.21D+05	-0.001131
256	0	22	2	23	-0.6977917D-02	-0.6986278D-02	0.8361364D-05	0.21D+05	-0.001198
257	0	23	2	24	-0.6998683D-02	-0.7007437D-02	0.8754394D-05	0.20D+05	-0.001251

258	0	24	2	25	-0.7019304D-02	-0.7028787D-02	0.9482996D-05	0.20D+05	-0.001351
259	0	25	2	26	-0.7040140D-02	-0.7050328D-02	0.1018762D-04	0.20D+05	-0.001447
260	0	26	2	27	-0.7061065D-02	-0.7072060D-02	0.1099473D-04	0.20D+05	-0.001557
261	0	27	2	28	-0.7082090D-02	-0.7093984D-02	0.1189382D-04	0.20D+05	-0.001679
262	0	28	2	29	-0.7103288D-02	-0.7116100D-02	0.1281235D-04	0.20D+05	-0.001804
263	0	29	2	30	-0.7124625D-02	-0.7138410D-02	0.1378485D-04	0.20D+05	-0.001935
264	0	30	2	31	-0.7146025D-02	-0.7160913D-02	0.1488781D-04	0.20D+05	-0.002083
265	0	1	2	0	-0.6539000D-02	-0.6534672D-02	-0.4327858D-05	0.23D+05	0.000662
266	0	2	2	1	-0.6508000D-02	-0.6518166D-02	0.1016592D-04	0.24D+05	-0.001562
267	0	3	2	2	-0.6495000D-02	-0.6501843D-02	0.6842733D-05	0.24D+05	-0.001054
268	0	4	2	3	-0.6481000D-02	-0.6485702D-02	0.4702442D-05	0.24D+05	-0.000726
269	0	5	2	4	-0.6463000D-02	-0.6469745D-02	0.6744943D-05	0.24D+05	-0.001044
270	0	6	2	5	-0.6452000D-02	-0.6453970D-02	0.1970118D-05	0.24D+05	-0.000305
271	0	7	2	6	-0.6446000D-02	-0.6438378D-02	-0.7622141D-05	0.24D+05	0.001182
272	0	8	2	7	-0.6418000D-02	-0.6422968D-02	0.4968104D-05	0.24D+05	-0.000774
273	0	9	2	8	-0.6404000D-02	-0.6407741D-02	0.3740761D-05	0.24D+05	-0.000584
274	0	10	2	9	-0.6387000D-02	-0.6392696D-02	0.5695779D-05	0.25D+05	-0.000892
275	0	11	2	10	-0.6370000D-02	-0.6377833D-02	0.7833105D-05	0.25D+05	-0.001230
276	0	12	2	11	-0.6360000D-02	-0.6363153D-02	0.3152708D-05	0.25D+05	-0.000496
277	0	13	2	12	-0.6341000D-02	-0.6348655D-02	0.7654556D-05	0.25D+05	-0.001207
278	0	14	2	13	-0.6327000D-02	-0.6334339D-02	0.7338629D-05	0.25D+05	-0.001160
279	0	15	2	14	-0.6311000D-02	-0.6320205D-02	0.9204937D-05	0.25D+05	-0.001459
280	0	16	2	15	-0.6300000D-02	-0.6306253D-02	0.6253487D-05	0.25D+05	-0.000993
281	0	17	2	16	-0.6295000D-02	-0.6292484D-02	-0.2515698D-05	0.25D+05	0.000400
282	0	18	2	17	-0.6272000D-02	-0.6278897D-02	0.6897413D-05	0.25D+05	-0.001100
283	0	19	2	18	-0.6263000D-02	-0.6265493D-02	0.2492857D-05	0.25D+05	-0.000398
284	0	20	2	19	-0.6225000D-02	-0.6252271D-02	0.2727071D-04	0.26D+05	-0.004381
285	0	21	2	20	-0.6213000D-02	-0.6239231D-02	0.2623103D-04	0.26D+05	-0.004222
286	0	22	2	21	-0.6172000D-02	-0.6226374D-02	0.5437390D-04	0.26D+05	-0.008810
287	0	23	2	22	-0.6232000D-02	-0.6213699D-02	-0.1830059D-04	0.26D+05	0.002937
288	0	24	2	23	-0.6109000D-02	-0.6201208D-02	0.9220769D-04	0.27D+05	-0.015094
289	0	25	2	24	-0.6267000D-02	-0.6188899D-02	-0.7810117D-04	0.25D+05	0.012462
290	0	0	2	1	-0.6566000D-02	-0.6568234D-02	0.2234262D-05	0.23D+05	-0.000340
291	0	1	2	2	-0.6579000D-02	-0.6585290D-02	0.6290490D-05	0.23D+05	-0.000956
292	0	2	2	3	-0.6595000D-02	-0.6602530D-02	0.7530386D-05	0.23D+05	-0.001142
293	0	3	2	4	-0.6616000D-02	-0.6619954D-02	0.3954150D-05	0.23D+05	-0.000598
294	0	4	2	5	-0.6629000D-02	-0.6637562D-02	0.8561978D-05	0.23D+05	-0.001292
295	0	5	2	6	-0.6659000D-02	-0.6655354D-02	-0.3645906D-05	0.23D+05	0.000548
296	0	6	2	7	-0.6669000D-02	-0.6673331D-02	0.4330718D-05	0.22D+05	-0.000649
297	0	7	2	8	-0.6687000D-02	-0.6691492D-02	0.4492075D-05	0.22D+05	-0.000672
298	0	8	2	9	-0.6709000D-02	-0.6709838D-02	0.8384228D-06	0.22D+05	-0.000125
299	0	9	2	10	-0.6726000D-02	-0.6728370D-02	0.2370016D-05	0.22D+05	-0.000352
300	0	10	2	11	-0.6748000D-02	-0.6747087D-02	-0.9128606D-06	0.22D+05	0.000135
301	0	11	2	12	-0.6758000D-02	-0.6765990D-02	0.7990063D-05	0.22D+05	-0.001182
302	0	12	2	13	-0.6792000D-02	-0.6785079D-02	-0.6920906D-05	0.22D+05	0.001019
303	0	13	2	14	-0.6805000D-02	-0.6804355D-02	-0.6454706D-06	0.22D+05	0.000095
304	0	14	2	15	-0.6818000D-02	-0.6823817D-02	0.5816688D-05	0.22D+05	-0.000853
305	0	15	2	16	-0.6839000D-02	-0.6843466D-02	0.4465905D-05	0.21D+05	-0.000653
306	0	16	2	17	-0.6860000D-02	-0.6863303D-02	0.3302528D-05	0.21D+05	-0.000481
307	0	17	2	18	-0.6859000D-02	-0.6883327D-02	0.2432691D-04	0.21D+05	-0.003547
308	0	18	2	19	-0.6883000D-02	-0.6903539D-02	0.2053941D-04	0.21D+05	-0.002984
309	0	19	2	20	-0.6918000D-02	-0.6923940D-02	0.5940399D-05	0.21D+05	-0.000859
310	0	20	2	21	-0.6950000D-02	-0.6944530D-02	-0.5469704D-05	0.21D+05	0.000787
311	0	21	2	22	-0.6961000D-02	-0.6965309D-02	0.4309471D-05	0.21D+05	-0.000619
312	0	22	2	23	-0.6951000D-02	-0.6986278D-02	0.3527836D-04	0.21D+05	-0.005075
313	0	23	2	24	-0.6897000D-02	-0.7007437D-02	0.1104374D-03	0.21D+05	-0.016012
314	0	24	2	25	-0.7008000D-02	-0.7028787D-02	0.2078700D-04	0.20D+05	-0.002966
315	0	25	2	26	-0.6889000D-02	-0.7050328D-02	0.1613276D-03	0.21D+05	-0.023418
316	0	26	2	27	-0.7106000D-02	-0.7072060D-02	-0.3394027D-04	0.20D+05	0.004776
317	0	27	2	28	-0.7226000D-02	-0.7093984D-02	-0.1320162D-03	0.19D+05	0.018270
318	0	28	2	29	-0.7202000D-02	-0.7116100D-02	-0.8589965D-04	0.19D+05	0.011927
319	0	1	2	0	-0.6509644D-02	-0.6534672D-02	0.2502814D-04	0.24D+05	-0.003845
320	0	2	2	1	-0.6494680D-02	-0.6518166D-02	0.2348592D-04	0.24D+05	-0.003616
321	0	3	2	2	-0.6472336D-02	-0.6501843D-02	0.2950673D-04	0.24D+05	-0.004559
322	0	4	2	3	-0.6448813D-02	-0.6485702D-02	0.3688944D-04	0.24D+05	-0.005720
323	0	5	2	4	-0.6436632D-02	-0.6469745D-02	0.3311294D-04	0.24D+05	-0.005144
324	0	6	2	5	-0.6404703D-02	-0.6453970D-02	0.4926712D-04	0.24D+05	-0.007692
325	0	7	2	6	-0.6385532D-02	-0.6438378D-02	0.5284586D-04	0.25D+05	-0.008276
326	0	8	2	7	-0.6373350D-02	-0.6422968D-02	0.4961810D-04	0.25D+05	-0.007785
327	0	9	2	8	-0.6370906D-02	-0.6407741D-02	0.3683476D-04	0.25D+05	-0.005782
328	0	10	2	9	-0.6354909D-02	-0.6392696D-02	0.3778678D-04	0.25D+05	-0.005946

329	0	11	2	10	-0.6346736D-02	-0.6377833D-02	0.3109710D-04	0.25D+05	-0.004900
330	0	12	2	11	-0.6341560D-02	-0.6363153D-02	0.2159271D-04	0.25D+05	-0.003405
331	0	13	2	12	-0.6337288D-02	-0.6348655D-02	0.1136656D-04	0.25D+05	-0.001794
332	0	14	2	13	-0.6324435D-02	-0.6334339D-02	0.9903629D-05	0.25D+05	-0.001566
333	0	15	2	14	-0.6318362D-02	-0.6320205D-02	0.1842937D-05	0.25D+05	-0.000292
334	0	16	2	15	-0.6304449D-02	-0.6306253D-02	0.1804487D-05	0.25D+05	-0.000286
335	0	17	2	16	-0.6296589D-02	-0.6292484D-02	-0.4104698D-05	0.25D+05	0.000652
336	0	18	2	17	-0.6286834D-02	-0.6278897D-02	-0.7936587D-05	0.25D+05	0.001262
337	0	19	2	18	-0.6290862D-02	-0.6265493D-02	-0.2536914D-04	0.25D+05	0.004033
338	0	20	2	19	-0.6277121D-02	-0.6252271D-02	-0.2485029D-04	0.25D+05	0.003959
339	0	21	2	20	-0.6263546D-02	-0.6239231D-02	-0.2431497D-04	0.25D+05	0.003882
340	0	22	2	21	-0.6248688D-02	-0.6226374D-02	-0.2231410D-04	0.26D+05	0.003571
341	0	23	2	22	-0.6227481D-02	-0.6213699D-02	-0.1378159D-04	0.26D+05	0.002213
342	0	24	2	23	-0.6259951D-02	-0.6201208D-02	-0.5874331D-04	0.26D+05	0.009384
343	0	0	2	1	-0.6552796D-02	-0.6568234D-02	0.1543826D-04	0.23D+05	-0.002356
344	0	1	2	2	-0.6542106D-02	-0.6585290D-02	0.4318449D-04	0.23D+05	-0.006601
345	0	2	2	3	-0.6570107D-02	-0.6602530D-02	0.3242339D-04	0.23D+05	-0.004935
346	0	3	2	4	-0.6578867D-02	-0.6619954D-02	0.4108715D-04	0.23D+05	-0.006245
347	0	4	2	5	-0.6582816D-02	-0.6637562D-02	0.5474598D-04	0.23D+05	-0.008316
348	0	5	2	6	-0.6587334D-02	-0.6655354D-02	0.6802009D-04	0.23D+05	-0.010326
349	0	6	2	7	-0.6595300D-02	-0.6673331D-02	0.7803072D-04	0.23D+05	-0.011831
350	0	7	2	8	-0.6606839D-02	-0.6691492D-02	0.8465308D-04	0.23D+05	-0.012813
351	0	8	2	9	-0.6632258D-02	-0.6709838D-02	0.7758042D-04	0.23D+05	-0.011697
352	0	9	2	10	-0.6657447D-02	-0.6728370D-02	0.7092302D-04	0.23D+05	-0.010653
353	0	10	2	11	-0.6683725D-02	-0.6747087D-02	0.6336214D-04	0.22D+05	-0.009480
354	0	11	2	12	-0.6712133D-02	-0.6765990D-02	0.5385706D-04	0.22D+05	-0.008024
355	0	12	2	13	-0.6746816D-02	-0.6785079D-02	0.3826309D-04	0.22D+05	-0.005671
356	0	13	2	14	-0.6780162D-02	-0.6804355D-02	0.2419253D-04	0.22D+05	-0.003568
357	0	14	2	15	-0.6807836D-02	-0.6823817D-02	0.1598069D-04	0.22D+05	-0.002347
358	0	15	2	16	-0.6837811D-02	-0.6843466D-02	0.5654905D-05	0.21D+05	-0.000827
359	0	16	2	17	-0.6863071D-02	-0.6863303D-02	0.2315283D-06	0.21D+05	-0.000034
360	0	17	2	18	-0.6890698D-02	-0.6883327D-02	-0.7371090D-05	0.21D+05	0.001070
361	0	18	2	19	-0.6912000D-02	-0.6903539D-02	-0.8460590D-05	0.21D+05	0.001224
362	0	19	2	20	-0.6936000D-02	-0.6923940D-02	-0.1205960D-04	0.21D+05	0.001739
363	0	20	2	21	-0.6961000D-02	-0.6944530D-02	-0.1646970D-04	0.21D+05	0.002366
364	0	21	2	22	-0.6985000D-02	-0.6965309D-02	-0.1969053D-04	0.20D+05	0.002819
365	0	22	2	23	-0.6999000D-02	-0.6986278D-02	-0.1272164D-04	0.20D+05	0.001818
366	0	23	2	24	-0.7024000D-02	-0.7007437D-02	-0.1656261D-04	0.20D+05	0.002358
367	0	1	2	0	-0.6548495D-02	-0.6534672D-02	-0.1382286D-04	0.23D+05	0.002111
368	0	2	2	1	-0.6530991D-02	-0.6518166D-02	-0.1282508D-04	0.23D+05	0.001964
369	0	3	2	2	-0.6505666D-02	-0.6501843D-02	-0.3823267D-05	0.24D+05	0.000588
370	0	4	2	3	-0.6488030D-02	-0.6485702D-02	-0.2327558D-05	0.24D+05	0.000359
371	0	5	2	4	-0.6470316D-02	-0.6469745D-02	-0.5710567D-06	0.24D+05	0.000088
372	0	6	2	5	-0.6455899D-02	-0.6453970D-02	-0.1928882D-05	0.24D+05	0.000299
373	0	7	2	6	-0.6444887D-02	-0.6438378D-02	-0.6509141D-05	0.24D+05	0.001010
374	0	8	2	7	-0.6436001D-02	-0.6422968D-02	-0.1303290D-04	0.24D+05	0.002025
375	0	9	2	8	-0.6422560D-02	-0.6407741D-02	-0.1481924D-04	0.24D+05	0.002307
376	0	10	2	9	-0.6422377D-02	-0.6392696D-02	-0.2968122D-04	0.24D+05	0.004622
377	0	11	2	10	-0.6403056D-02	-0.6377833D-02	-0.2522290D-04	0.24D+05	0.003939
378	0	12	2	11	-0.6390858D-02	-0.6363153D-02	-0.2770529D-04	0.24D+05	0.004335
379	0	13	2	12	-0.6389615D-02	-0.6348655D-02	-0.4096044D-04	0.24D+05	0.006410
380	0	14	2	13	-0.6335469D-02	-0.6334339D-02	-0.1130371D-05	0.25D+05	0.000178
381	0	15	2	14	-0.6348416D-02	-0.6320205D-02	-0.2821106D-04	0.25D+05	0.004444
382	0	16	2	15	-0.6338139D-02	-0.6306253D-02	-0.3188551D-04	0.25D+05	0.005031
383	0	17	2	16	-0.6335270D-02	-0.6292484D-02	-0.4278570D-04	0.25D+05	0.006754
384	0	18	2	17	-0.6339010D-02	-0.6278897D-02	-0.6011259D-04	0.25D+05	0.009483
385	0	19	2	18	-0.6316761D-02	-0.6265493D-02	-0.5126814D-04	0.25D+05	0.008116
386	0	20	2	19	-0.6290388D-02	-0.6252271D-02	-0.3811729D-04	0.25D+05	0.006060
387	0	21	2	20	-0.6280757D-02	-0.6239231D-02	-0.4152597D-04	0.25D+05	0.006612
388	0	22	2	21	-0.6285487D-02	-0.6226374D-02	-0.5911310D-04	0.25D+05	0.009405
389	0	23	2	22	-0.6295152D-02	-0.6213699D-02	-0.8145259D-04	0.25D+05	0.012939
390	0	24	2	23	-0.6290130D-02	-0.6201208D-02	-0.8892231D-04	0.25D+05	0.014137
391	0	0	2	1	-0.6571091D-02	-0.6568234D-02	-0.2856738D-05	0.23D+05	0.000435
392	0	1	2	2	-0.6588562D-02	-0.6585290D-02	-0.3271510D-05	0.23D+05	0.000497
393	0	2	2	3	-0.6603034D-02	-0.6602530D-02	-0.5036144D-06	0.23D+05	0.000076
394	0	3	2	4	-0.6617146D-02	-0.6619954D-02	0.2808150D-05	0.23D+05	-0.000424
395	0	4	2	5	-0.6633941D-02	-0.6637562D-02	0.3620978D-05	0.23D+05	-0.000546
396	0	5	2	6	-0.6656336D-02	-0.6655354D-02	-0.9819061D-06	0.23D+05	0.000148
397	0	6	2	7	-0.6677999D-02	-0.6673331D-02	-0.4668282D-05	0.22D+05	0.000699
398	0	7	2	8	-0.6700754D-02	-0.6691492D-02	-0.9261925D-05	0.22D+05	0.001382
399	0	8	2	9	-0.6719116D-02	-0.6709838D-02	-0.9277577D-05	0.22D+05	0.001381

400	0	9	2	10	-0.6744079D-02	-0.6728370D-02	-0.1570898D-04	0.22D+05	0.002329
401	0	10	2	11	-0.6765195D-02	-0.6747087D-02	-0.1810786D-04	0.22D+05	0.002677
402	0	11	2	12	-0.6788715D-02	-0.6765990D-02	-0.2272494D-04	0.22D+05	0.003347
403	0	12	2	13	-0.6814911D-02	-0.6785079D-02	-0.2983191D-04	0.22D+05	0.004377
404	0	13	2	14	-0.6837257D-02	-0.6804355D-02	-0.3290247D-04	0.21D+05	0.004812
405	0	14	2	15	-0.6854314D-02	-0.6823817D-02	-0.3049731D-04	0.21D+05	0.004449
406	0	15	2	16	-0.6879765D-02	-0.6843466D-02	-0.3629909D-04	0.21D+05	0.005276
407	0	16	2	17	-0.6906465D-02	-0.6863303D-02	-0.4316247D-04	0.21D+05	0.006250
408	0	17	2	18	-0.6934785D-02	-0.6883327D-02	-0.5145809D-04	0.21D+05	0.007420
409	0	18	2	19	-0.6961155D-02	-0.6903539D-02	-0.5761559D-04	0.21D+05	0.008277
410	0	19	2	20	-0.6962356D-02	-0.6923940D-02	-0.3841560D-04	0.21D+05	0.005518
411	0	20	2	21	-0.6997544D-02	-0.6944530D-02	-0.5301370D-04	0.20D+05	0.007576
412	0	21	2	22	-0.7011994D-02	-0.6965309D-02	-0.4668453D-04	0.20D+05	0.006658
413	0	22	2	23	-0.7062609D-02	-0.6986278D-02	-0.7633064D-04	0.20D+05	0.010808
414	0	26	3	25	-0.3614961D-03	-0.3622265D-03	0.7303673D-06	0.77D+07	-0.002020
415	0	27	3	26	-0.3604511D-03	-0.3604309D-03	-0.2017407D-07	0.77D+07	0.000056
416	0	28	3	27	-0.3589984D-03	-0.3586597D-03	-0.3387057D-06	0.78D+07	0.000943
417	0	27	3	26	-0.3558645D-03	-0.3604309D-03	0.4566426D-05	0.79D+07	-0.012832
418	0	28	3	27	-0.3532651D-03	-0.3586597D-03	0.5394594D-05	0.80D+07	-0.015271
419	0	1	3	0	-0.4112259D-03	-0.4152257D-03	0.3999818D-05	0.59D+07	-0.009727
420	0	2	3	1	-0.4099018D-03	-0.4127971D-03	0.2895327D-05	0.60D+07	-0.007063
421	0	3	3	2	-0.4071356D-03	-0.4103951D-03	0.3259502D-05	0.60D+07	-0.008006
422	0	4	3	3	-0.4048075D-03	-0.4080195D-03	0.3212010D-05	0.61D+07	-0.007935
423	0	5	3	4	-0.4009313D-03	-0.4056702D-03	0.4738923D-05	0.62D+07	-0.011820
424	0	6	3	5	-0.3986529D-03	-0.4033471D-03	0.4694238D-05	0.63D+07	-0.011775
425	0	7	3	6	-0.3973720D-03	-0.4010501D-03	0.3678126D-05	0.63D+07	-0.009256
426	0	8	3	7	-0.3957276D-03	-0.3987791D-03	0.3051467D-05	0.64D+07	-0.007711
427	0	9	3	8	-0.3931544D-03	-0.3965339D-03	0.3379452D-05	0.65D+07	-0.008596
428	0	10	3	9	-0.3916904D-03	-0.3943144D-03	0.2623976D-05	0.65D+07	-0.006699
429	0	11	3	10	-0.3887344D-03	-0.3921205D-03	0.3386130D-05	0.66D+07	-0.008711
430	0	12	3	11	-0.3854260D-03	-0.3899522D-03	0.4526216D-05	0.67D+07	-0.011743
431	0	13	3	12	-0.3851399D-03	-0.3878093D-03	0.2669428D-05	0.67D+07	-0.006931
432	0	14	3	13	-0.3838433D-03	-0.3856918D-03	0.1848473D-05	0.68D+07	-0.004816
433	0	15	3	14	-0.3817532D-03	-0.3835995D-03	0.1846269D-05	0.69D+07	-0.004836
434	0	16	3	15	-0.3806651D-03	-0.3815323D-03	0.8672159D-06	0.69D+07	-0.002278
435	0	17	3	16	-0.3784964D-03	-0.3794902D-03	0.9938141D-06	0.70D+07	-0.002626
436	0	18	3	17	-0.3819037D-03	-0.3774731D-03	-0.4430602D-05	0.69D+07	0.011601
437	0	0	3	1	-0.4139409D-03	-0.4201631D-03	0.6222230D-05	0.58D+07	-0.015032
438	0	1	3	2	-0.4171083D-03	-0.4226722D-03	0.5563917D-05	0.57D+07	-0.013339
439	0	2	3	3	-0.4206975D-03	-0.4252084D-03	0.4510923D-05	0.57D+07	-0.010722
440	0	3	3	4	-0.4235302D-03	-0.4277719D-03	0.4241673D-05	0.56D+07	-0.010015
441	0	4	3	5	-0.4266929D-03	-0.4303627D-03	0.3669828D-05	0.55D+07	-0.008601
442	0	5	3	6	-0.4288828D-03	-0.4329811D-03	0.4098339D-05	0.54D+07	-0.009556
443	0	6	3	7	-0.4318629D-03	-0.4356273D-03	0.3764363D-05	0.54D+07	-0.008717
444	0	7	3	8	-0.4338359D-03	-0.4383013D-03	0.4465355D-05	0.53D+07	-0.010293
445	0	8	3	9	-0.4373442D-03	-0.4410033D-03	0.3659073D-05	0.52D+07	-0.008367
446	0	9	3	10	-0.4399884D-03	-0.4437335D-03	0.3745088D-05	0.52D+07	-0.008512
447	0	10	3	11	-0.4422255D-03	-0.4464921D-03	0.4266570D-05	0.51D+07	-0.009648
448	0	11	3	12	-0.4451004D-03	-0.4492792D-03	0.4178786D-05	0.50D+07	-0.009388
449	0	12	3	13	-0.4479872D-03	-0.4520950D-03	0.4107821D-05	0.50D+07	-0.009170
450	0	13	3	14	-0.4500924D-03	-0.4549397D-03	0.4847349D-05	0.49D+07	-0.010770
451	0	14	3	15	-0.4540166D-03	-0.4578136D-03	0.3796952D-05	0.49D+07	-0.008363
452	0	15	3	16	-0.4576110D-03	-0.4607166D-03	0.3105632D-05	0.48D+07	-0.006787
453	0	16	3	17	-0.4608004D-03	-0.4636492D-03	0.2848766D-05	0.47D+07	-0.006182
454	0	17	3	18	-0.4635997D-03	-0.4666113D-03	0.3011645D-05	0.47D+07	-0.006496
455	0	18	3	19	-0.4647795D-03	-0.4696034D-03	0.4823879D-05	0.46D+07	-0.010379
456	0	19	3	20	-0.4692667D-03	-0.4726255D-03	0.3358769D-05	0.45D+07	-0.007157
457	0	20	3	21	-0.4696943D-03	-0.4756778D-03	0.5983524D-05	0.45D+07	-0.012739
458	0	21	3	22	-0.4714473D-03	-0.4787607D-03	0.7313351D-05	0.45D+07	-0.015513
459	0	22	3	23	-0.4728004D-03	-0.4818742D-03	0.9073769D-05	0.45D+07	-0.019192
460	0	1	3	0	-0.4088299D-03	-0.4152257D-03	0.6395818D-05	0.60D+07	-0.015644
461	0	2	3	1	-0.4066049D-03	-0.4127971D-03	0.6192227D-05	0.60D+07	-0.015229
462	0	3	3	2	-0.4038860D-03	-0.4103951D-03	0.6509102D-05	0.61D+07	-0.016116
463	0	4	3	3	-0.4022376D-03	-0.4080195D-03	0.5781910D-05	0.62D+07	-0.014374
464	0	5	3	4	-0.3996388D-03	-0.4056702D-03	0.6031423D-05	0.63D+07	-0.015092
465	0	6	3	5	-0.3977052D-03	-0.4033471D-03	0.5641938D-05	0.63D+07	-0.014186
466	0	7	3	6	-0.3957543D-03	-0.4010501D-03	0.5295826D-05	0.64D+07	-0.013382
467	0	8	3	7	-0.3939463D-03	-0.3987791D-03	0.4832767D-05	0.64D+07	-0.012268
468	0	9	3	8	-0.3907069D-03	-0.3965339D-03	0.5826952D-05	0.66D+07	-0.014914
469	0	10	3	9	-0.3896396D-03	-0.3943144D-03	0.4674776D-05	0.66D+07	-0.011998
470	0	11	3	10	-0.3875581D-03	-0.3921205D-03	0.4562430D-05	0.67D+07	-0.011772

471	0	12	3	11	-0.3853229D-03	-0.3899522D-03	0.4629316D-05	0.67D+07	-0.012014
472	0	13	3	12	-0.3841081D-03	-0.3878093D-03	0.3701228D-05	0.68D+07	-0.009636
473	0	14	3	13	-0.3812133D-03	-0.3856918D-03	0.4478473D-05	0.69D+07	-0.011748
474	0	15	3	14	-0.3792630D-03	-0.3835995D-03	0.4336469D-05	0.70D+07	-0.011434
475	0	16	3	15	-0.3776662D-03	-0.3815323D-03	0.3866116D-05	0.70D+07	-0.010237
476	0	17	3	16	-0.3759870D-03	-0.3794902D-03	0.3503214D-05	0.71D+07	-0.009317
477	0	18	3	17	-0.3735003D-03	-0.3774731D-03	0.3972798D-05	0.72D+07	-0.010637
478	0	19	3	18	-0.3727079D-03	-0.3754809D-03	0.2772983D-05	0.72D+07	-0.007440
479	0	20	3	19	-0.3686832D-03	-0.3735135D-03	0.4830291D-05	0.74D+07	-0.013101
480	0	0	3	1	-0.4143347D-03	-0.4201631D-03	0.5828430D-05	0.58D+07	-0.014067
481	0	1	3	2	-0.4165985D-03	-0.4226722D-03	0.6073717D-05	0.58D+07	-0.014579
482	0	2	3	3	-0.4188377D-03	-0.4252084D-03	0.6370723D-05	0.57D+07	-0.015210
483	0	3	3	4	-0.4220922D-03	-0.4277719D-03	0.5679673D-05	0.56D+07	-0.013456
484	0	4	3	5	-0.4245619D-03	-0.4303627D-03	0.5800828D-05	0.55D+07	-0.013663
485	0	5	3	6	-0.4266701D-03	-0.4329811D-03	0.6311039D-05	0.55D+07	-0.014791
486	0	6	3	7	-0.4299814D-03	-0.4356273D-03	0.5645863D-05	0.54D+07	-0.013130
487	0	7	3	8	-0.4323035D-03	-0.4383013D-03	0.5997755D-05	0.54D+07	-0.013874
488	0	8	3	9	-0.4350201D-03	-0.4410033D-03	0.5983173D-05	0.53D+07	-0.013754
489	0	9	3	10	-0.4380770D-03	-0.4437335D-03	0.5656488D-05	0.52D+07	-0.012912
490	0	10	3	11	-0.4411299D-03	-0.4464921D-03	0.5362170D-05	0.51D+07	-0.012156
491	0	11	3	12	-0.4435340D-03	-0.4492792D-03	0.5745186D-05	0.51D+07	-0.012953
492	0	12	3	13	-0.4472957D-03	-0.4520950D-03	0.4799321D-05	0.50D+07	-0.010730
493	0	13	3	14	-0.4507088D-03	-0.4549397D-03	0.4230949D-05	0.49D+07	-0.009387
494	0	14	3	15	-0.4532482D-03	-0.4578136D-03	0.4565352D-05	0.49D+07	-0.010073
495	0	15	3	16	-0.4559249D-03	-0.4607166D-03	0.4791732D-05	0.48D+07	-0.010510
496	0	16	3	17	-0.4592336D-03	-0.4636492D-03	0.4415566D-05	0.47D+07	-0.009615
497	0	17	3	18	-0.4610637D-03	-0.4666113D-03	0.5547645D-05	0.47D+07	-0.012032
498	0	18	3	19	-0.4632980D-03	-0.4696034D-03	0.6305379D-05	0.47D+07	-0.013610
499	0	19	3	20	-0.4671759D-03	-0.4726255D-03	0.5449569D-05	0.46D+07	-0.011665
500	0	20	3	21	-0.4715534D-03	-0.4756778D-03	0.4124424D-05	0.45D+07	-0.008746
501	0	1	3	0	-0.4147955D-03	-0.4152257D-03	0.4302180D-06	0.58D+07	-0.001037
502	0	2	3	1	-0.4117223D-03	-0.4127971D-03	0.1074827D-05	0.59D+07	-0.002611
503	0	3	3	2	-0.4114359D-03	-0.4103951D-03	-0.1040798D-05	0.59D+07	0.002530
504	0	4	3	3	-0.4074637D-03	-0.4080195D-03	0.5558103D-06	0.60D+07	-0.001364
505	0	5	3	4	-0.4048062D-03	-0.4056702D-03	0.8640225D-06	0.61D+07	-0.002134
506	0	6	3	5	-0.4026775D-03	-0.4033471D-03	0.6696383D-06	0.62D+07	-0.001663
507	0	7	3	6	-0.3995175D-03	-0.4010501D-03	0.1532626D-05	0.63D+07	-0.003836
508	0	8	3	7	-0.3972795D-03	-0.3987791D-03	0.1499567D-05	0.63D+07	-0.003775
509	0	9	3	8	-0.3947839D-03	-0.3965339D-03	0.1749952D-05	0.64D+07	-0.004433
510	0	10	3	9	-0.3933399D-03	-0.3943144D-03	0.9744762D-06	0.65D+07	-0.002477
511	0	11	3	10	-0.3917121D-03	-0.3921205D-03	0.4084298D-06	0.65D+07	-0.001043
512	0	12	3	11	-0.3887788D-03	-0.3899522D-03	0.1173416D-05	0.66D+07	-0.003018
513	0	13	3	12	-0.3867644D-03	-0.3878093D-03	0.1044928D-05	0.67D+07	-0.002702
514	0	14	3	13	-0.3847157D-03	-0.3856918D-03	0.9760726D-06	0.68D+07	-0.002537
515	0	15	3	14	-0.3842889D-03	-0.3835995D-03	-0.6894312D-06	0.68D+07	0.001794
516	0	16	3	15	-0.3805836D-03	-0.3815323D-03	0.9487159D-06	0.69D+07	-0.002493
517	0	17	3	16	-0.3790979D-03	-0.3794902D-03	0.3923141D-06	0.70D+07	-0.001035
518	0	18	3	17	-0.3768872D-03	-0.3774731D-03	0.5858979D-06	0.70D+07	-0.001555
519	0	19	3	18	-0.3743426D-03	-0.3754809D-03	0.1138283D-05	0.71D+07	-0.003041
520	0	20	3	19	-0.3722386D-03	-0.3735135D-03	0.1274891D-05	0.72D+07	-0.003425
521	0	21	3	20	-0.3736188D-03	-0.3715708D-03	-0.2047950D-05	0.72D+07	0.005481
522	0	22	3	21	-0.3643772D-03	-0.3696529D-03	0.5275682D-05	0.75D+07	-0.014479
523	0	23	3	22	-0.3626713D-03	-0.3677595D-03	0.5088234D-05	0.76D+07	-0.014030
524	0	24	3	23	-0.3751095D-03	-0.3658907D-03	-0.9218786D-05	0.71D+07	0.024576
525	0	25	3	24	-0.3660204D-03	-0.3640464D-03	-0.1974011D-05	0.75D+07	0.005393
526	0	0	3	1	-0.4192734D-03	-0.4201631D-03	0.8897300D-06	0.57D+07	-0.002122
527	0	1	3	2	-0.4228409D-03	-0.4226722D-03	-0.1686830D-06	0.56D+07	0.000399
528	0	2	3	3	-0.4257044D-03	-0.4252084D-03	-0.4959773D-06	0.55D+07	0.001165
529	0	3	3	4	-0.4276383D-03	-0.4277719D-03	0.1335729D-06	0.55D+07	-0.000312
530	0	4	3	5	-0.4290867D-03	-0.4303627D-03	0.1276028D-05	0.54D+07	-0.002974
531	0	5	3	6	-0.4308947D-03	-0.4329811D-03	0.2086439D-05	0.54D+07	-0.004842
532	0	6	3	7	-0.4329989D-03	-0.4356273D-03	0.2628363D-05	0.53D+07	-0.006070
533	0	7	3	8	-0.4346985D-03	-0.4383013D-03	0.3602755D-05	0.53D+07	-0.008288
534	0	8	3	9	-0.4367608D-03	-0.4410033D-03	0.4242473D-05	0.52D+07	-0.009713
535	0	9	3	10	-0.4413180D-03	-0.4437335D-03	0.2415488D-05	0.51D+07	-0.005473
536	0	10	3	11	-0.4443031D-03	-0.4464921D-03	0.2188970D-05	0.51D+07	-0.004927
537	0	11	3	12	-0.4460350D-03	-0.4492792D-03	0.3244186D-05	0.50D+07	-0.007273
538	0	12	3	13	-0.4515071D-03	-0.4520950D-03	0.5879213D-06	0.49D+07	-0.001302
539	0	13	3	14	-0.4533627D-03	-0.4549397D-03	0.1577049D-05	0.49D+07	-0.003479
540	0	14	3	15	-0.4575042D-03	-0.4578136D-03	0.3093521D-06	0.48D+07	-0.000676
541	0	15	3	16	-0.4607485D-03	-0.4607166D-03	-0.3186818D-07	0.47D+07	0.000069

542	0	16	3	17	-0.4628363D-03	-0.4636492D-03	0.8128656D-06	0.47D+07	-0.001756
543	0	17	3	18	-0.4663892D-03	-0.4666113D-03	0.2221449D-06	0.46D+07	-0.000476
544	0	18	3	19	-0.4691372D-03	-0.4696034D-03	0.4661791D-06	0.45D+07	-0.000994
545	0	19	3	20	-0.4731294D-03	-0.4726255D-03	-0.5039311D-06	0.45D+07	0.001065
546	0	20	3	21	-0.4748655D-03	-0.4756778D-03	0.8123236D-06	0.44D+07	-0.001711
547	0	21	3	22	-0.4799443D-03	-0.4787607D-03	-0.1183649D-05	0.43D+07	0.002466
548	0	22	3	23	-0.4792684D-03	-0.4818742D-03	0.2605769D-05	0.44D+07	-0.005437
549	0	23	3	24	-0.4810550D-03	-0.4850186D-03	0.3963601D-05	0.43D+07	-0.008239
550	0	24	3	25	-0.4851646D-03	-0.4881941D-03	0.3029547D-05	0.42D+07	-0.006244
551	0	25	3	26	-0.4852487D-03	-0.4914011D-03	0.6152371D-05	0.42D+07	-0.012679
552	0	1	3	0	-0.4124095D-03	-0.4152257D-03	0.2816218D-05	0.59D+07	-0.006829
553	0	12	3	11	-0.3822253D-03	-0.3899522D-03	0.7726916D-05	0.68D+07	-0.020216
554	0	13	3	12	-0.3790633D-03	-0.3878093D-03	0.8746028D-05	0.70D+07	-0.023073
555	0	14	3	13	-0.3781542D-03	-0.3856918D-03	0.7537573D-05	0.70D+07	-0.019933
556	0	15	3	14	-0.3771931D-03	-0.3835995D-03	0.6406369D-05	0.70D+07	-0.016984
557	0	16	3	15	-0.3785402D-03	-0.3815323D-03	0.2992116D-05	0.70D+07	-0.007904
558	0	17	3	16	-0.3759879D-03	-0.3794902D-03	0.3502314D-05	0.71D+07	-0.009315
559	0	18	3	17	-0.3750103D-03	-0.3774731D-03	0.2462798D-05	0.71D+07	-0.006567
560	0	19	3	18	-0.3747512D-03	-0.3754809D-03	0.7296829D-06	0.71D+07	-0.001947
561	0	20	3	19	-0.3743568D-03	-0.3735135D-03	-0.8433089D-06	0.71D+07	0.002253
562	0	21	3	20	-0.3727044D-03	-0.3715708D-03	-0.1133550D-05	0.72D+07	0.003041
563	0	22	3	21	-0.3714127D-03	-0.3696529D-03	-0.1759818D-05	0.72D+07	0.004738
564	0	23	3	22	-0.3701778D-03	-0.3677595D-03	-0.2418266D-05	0.73D+07	0.006533
565	0	24	3	23	-0.3684546D-03	-0.3658907D-03	-0.2563886D-05	0.74D+07	0.006958
566	0	25	3	24	-0.3663176D-03	-0.3640464D-03	-0.2271211D-05	0.75D+07	0.006200
567	0	26	3	25	-0.3645991D-03	-0.3622265D-03	-0.2372633D-05	0.75D+07	0.006508
568	0	27	3	26	-0.3634260D-03	-0.3604309D-03	-0.2995074D-05	0.76D+07	0.008241
569	0	28	3	27	-0.3630368D-03	-0.3586597D-03	-0.4377106D-05	0.76D+07	0.012057
570	0	29	3	28	-0.3605274D-03	-0.3569127D-03	-0.3614681D-05	0.77D+07	0.010026
571	0	30	3	29	-0.3591904D-03	-0.3551900D-03	-0.4000430D-05	0.78D+07	0.011137
572	0	31	3	30	-0.3576381D-03	-0.3534914D-03	-0.4146713D-05	0.78D+07	0.011595
573	0	32	3	31	-0.3554263D-03	-0.3518169D-03	-0.3609371D-05	0.79D+07	0.010155
574	0	33	3	32	-0.3539993D-03	-0.3501666D-03	-0.3832725D-05	0.80D+07	0.010827
575	0	35	3	34	-0.3489244D-03	-0.3469380D-03	-0.1986412D-05	0.82D+07	0.005693
576	0	36	3	35	-0.3471033D-03	-0.3453597D-03	-0.1743588D-05	0.83D+07	0.005023
577	0	37	3	36	-0.3461024D-03	-0.3438054D-03	-0.2296994D-05	0.83D+07	0.006637
578	0	38	3	37	-0.3253236D-03	-0.3422751D-03	0.1695146D-04	0.94D+07	-0.052106
579	0	39	3	38	-0.3472254D-03	-0.3407686D-03	-0.6456780D-05	0.83D+07	0.018595
580	0	0	3	1	-0.4168115D-03	-0.4201631D-03	0.3351630D-05	0.58D+07	-0.008041
581	0	15	3	16	-0.4495562D-03	-0.4607166D-03	0.1116043D-04	0.49D+07	-0.024825
582	0	16	3	17	-0.4559043D-03	-0.4636492D-03	0.7744866D-05	0.48D+07	-0.016988
583	0	17	3	18	-0.4632023D-03	-0.4666113D-03	0.3409045D-05	0.47D+07	-0.007360
584	0	18	3	19	-0.4649911D-03	-0.4696034D-03	0.4612279D-05	0.46D+07	-0.009919
585	0	19	3	20	-0.4679351D-03	-0.4726255D-03	0.4690369D-05	0.46D+07	-0.010024
586	0	20	3	21	-0.4736277D-03	-0.4756778D-03	0.2050124D-05	0.45D+07	-0.004329
587	0	21	3	22	-0.4776357D-03	-0.4787607D-03	0.1124951D-05	0.44D+07	-0.002355
588	0	22	3	23	-0.4827905D-03	-0.4818742D-03	-0.9163308D-06	0.43D+07	0.001898
589	0	23	3	24	-0.4871390D-03	-0.4850186D-03	-0.2120399D-05	0.42D+07	0.004353
590	0	24	3	25	-0.4899459D-03	-0.4881941D-03	-0.1751753D-05	0.42D+07	0.003575
591	0	25	3	26	-0.4936405D-03	-0.4914011D-03	-0.2239429D-05	0.41D+07	0.004537
592	0	26	3	27	-0.4970825D-03	-0.4946396D-03	-0.2442932D-05	0.40D+07	0.004915
593	0	27	3	28	-0.5012841D-03	-0.4979099D-03	-0.3374202D-05	0.40D+07	0.006731
594	0	28	3	29	-0.5061250D-03	-0.5012123D-03	-0.4912700D-05	0.39D+07	0.009706
595	0	29	3	30	-0.5106333D-03	-0.5045470D-03	-0.6086290D-05	0.38D+07	0.011919
596	0	30	3	31	-0.5143140D-03	-0.5079143D-03	-0.6399717D-05	0.38D+07	0.012443
597	0	31	3	32	-0.5176228D-03	-0.5113144D-03	-0.6308426D-05	0.37D+07	0.012187
598	0	32	3	33	-0.5239672D-03	-0.5147475D-03	-0.9219668D-05	0.36D+07	0.017596
599	0	33	3	34	-0.5319712D-03	-0.5182140D-03	-0.1375717D-04	0.35D+07	0.025861
600	0	36	3	37	-0.5458906D-03	-0.5288162D-03	-0.1707435D-04	0.34D+07	0.031278
601	0	37	3	38	-0.5586814D-03	-0.5324188D-03	-0.2626258D-04	0.32D+07	0.047008
602	0	38	3	39	-0.5833331D-03	-0.5360561D-03	-0.4727699D-04	0.29D+07	0.081046
603	0	39	3	40	-0.6181495D-03	-0.5397284D-03	-0.7842108D-04	0.00D+00	0.126864
604	0	40	3	41	-0.5542196D-03	-0.5434360D-03	-0.1078355D-04	0.33D+07	0.019457
605	0	41	3	42	-0.6926667D-03	-0.5471793D-03	-0.1454874D-03	0.00D+00	0.210040
606	0	23	3	24	-0.4858141D-03	-0.4850186D-03	-0.7954994D-06	0.42D+07	0.001637
607	0	19	4	18	-0.1485932D-04	-0.1485821D-04	-0.1111682D-08	0.45D+10	0.000075
608	0	20	4	19	-0.1459058D-04	-0.1457651D-04	-0.1407486D-07	0.47D+10	0.000965
609	0	21	4	20	-0.1432917D-04	-0.1429778D-04	-0.3139474D-07	0.49D+10	0.002191
610	0	22	4	21	-0.1406711D-04	-0.1402202D-04	-0.4508710D-07	0.51D+10	0.003205
611	0	23	4	22	-0.1377741D-04	-0.1374923D-04	-0.2818324D-07	0.53D+10	0.002046
612	0	24	4	23	-0.1349306D-04	-0.1347938D-04	-0.1368457D-07	0.55D+10	0.001014

613	0	27	4	26	-0.1279671D-04	-0.1268725D-04	-0.1094626D-06	0.61D+10	0.008554
614	0	28	4	27	-0.1234285D-04	-0.1242897D-04	0.8611886D-07	0.66D+10	-0.006977
615	0	32	4	31	-0.1212537D-04	-0.1142418D-04	-0.7011884D-06	0.68D+10	0.057828
616	0	33	4	32	-0.1126493D-04	-0.1118000D-04	-0.8492991D-07	0.79D+10	0.007539
617	0	34	4	33	-0.1074870D-04	-0.1093859D-04	0.1898938D-06	0.87D+10	-0.017667
618	0	1	4	0	-0.2050145D-04	-0.2046752D-04	-0.3392712D-07	0.24D+10	0.001655
619	0	2	4	1	-0.1937941D-04	-0.2012781D-04	0.7484017D-06	0.27D+10	-0.038618
620	0	3	4	2	-0.1892510D-04	-0.1979157D-04	0.8664680D-06	0.28D+10	-0.045784
621	0	4	4	3	-0.1889875D-04	-0.1945873D-04	0.5599800D-06	0.28D+10	-0.029631
622	0	6	4	5	-0.1842745D-04	-0.1880323D-04	0.3757845D-06	0.29D+10	-0.020393
623	0	7	4	6	-0.1798792D-04	-0.1848050D-04	0.4925777D-06	0.31D+10	-0.027384
624	0	8	4	7	-0.1796301D-04	-0.1816107D-04	0.1980609D-06	0.31D+10	-0.011026
625	0	9	4	8	-0.1731321D-04	-0.1784493D-04	0.5317188D-06	0.33D+10	-0.030712
626	0	10	4	9	-0.1678084D-04	-0.1753203D-04	0.7511902D-06	0.36D+10	-0.044765
627	0	11	4	10	-0.1638027D-04	-0.1722236D-04	0.8420911D-06	0.37D+10	-0.051409
628	0	13	4	12	-0.1639199D-04	-0.1661260D-04	0.2206110D-06	0.37D+10	-0.013458
629	0	14	4	13	-0.1597767D-04	-0.1631246D-04	0.3347928D-06	0.39D+10	-0.020954
630	0	15	4	14	-0.1664812D-04	-0.1601546D-04	-0.6326650D-06	0.36D+10	0.038002
631	0	16	4	15	-0.1520743D-04	-0.1572155D-04	0.5141200D-06	0.43D+10	-0.033807
632	0	0	4	1	-0.2052111D-04	-0.2115743D-04	0.6363248D-06	0.24D+10	-0.031008
633	0	1	4	2	-0.2105775D-04	-0.2150770D-04	0.4499502D-06	0.23D+10	-0.021367
634	0	2	4	3	-0.2188380D-04	-0.2186155D-04	-0.2225133D-07	0.21D+10	0.001017
635	0	3	4	4	-0.2183165D-04	-0.2221903D-04	0.3873758D-06	0.21D+10	-0.017744
636	0	4	4	5	-0.2224372D-04	-0.2258014D-04	0.3364228D-06	0.20D+10	-0.015124
637	0	5	4	6	-0.2268686D-04	-0.2294497D-04	0.2581059D-06	0.19D+10	-0.011377
638	0	6	4	7	-0.2275649D-04	-0.2331351D-04	0.5570181D-06	0.19D+10	-0.024477
639	0	8	4	9	-0.2340555D-04	-0.2406191D-04	0.6563611D-06	0.18D+10	-0.028043
640	0	9	4	10	-0.2335506D-04	-0.2444186D-04	0.1086798D-05	0.18D+10	-0.046534
641	0	10	4	11	-0.2414686D-04	-0.2482568D-04	0.6788154D-06	0.17D+10	-0.028112
642	0	11	4	12	-0.2503544D-04	-0.2521341D-04	0.1779746D-06	0.16D+10	-0.007109
643	0	12	4	13	-0.2483436D-04	-0.2560512D-04	0.7707600D-06	0.16D+10	-0.031036
644	0	13	4	14	-0.2531737D-04	-0.2600082D-04	0.6834501D-06	0.16D+10	-0.026995
645	0	15	4	16	-0.2594066D-04	-0.2680440D-04	0.8637379D-06	0.15D+10	-0.033297
646	0	20	4	21	-0.2795203D-04	-0.2888643D-04	0.9343992D-06	0.13D+10	-0.033429
647	0	21	4	22	-0.2888777D-04	-0.2931572D-04	0.4279536D-06	0.12D+10	-0.014814
648	0	22	4	23	-0.2916510D-04	-0.2974944D-04	0.5843386D-06	0.12D+10	-0.020036
649	0	23	4	24	-0.3417056D-04	-0.3018762D-04	-0.3982944D-05	0.00D+00	0.116561
650	0	1	4	0	-0.2050125D-04	-0.2046752D-04	-0.3372712D-07	0.24D+10	0.001645
651	0	2	4	1	-0.2014692D-04	-0.2012781D-04	-0.1910826D-07	0.25D+10	0.000948
652	0	3	4	2	-0.1979723D-04	-0.1979157D-04	-0.5662021D-08	0.26D+10	0.000286
653	0	4	4	3	-0.1945244D-04	-0.1945873D-04	0.6290006D-08	0.26D+10	-0.000323
654	0	5	4	4	-0.1911281D-04	-0.1912930D-04	0.1649355D-07	0.27D+10	-0.000863
655	0	6	4	5	-0.1877863D-04	-0.1880323D-04	0.2460448D-07	0.28D+10	-0.001310
656	0	7	4	6	-0.1845020D-04	-0.1848050D-04	0.3029773D-07	0.29D+10	-0.001642
657	0	8	4	7	-0.1812781D-04	-0.1816107D-04	0.3326088D-07	0.30D+10	-0.001835
658	0	9	4	8	-0.1781180D-04	-0.1784493D-04	0.3312885D-07	0.32D+10	-0.001860
659	0	10	4	9	-0.1750252D-04	-0.1753203D-04	0.2951015D-07	0.33D+10	-0.001686
660	0	11	4	10	-0.1720033D-04	-0.1722236D-04	0.2203109D-07	0.34D+10	-0.001281
661	0	12	4	11	-0.1690561D-04	-0.1691590D-04	0.1028571D-07	0.35D+10	-0.000608
662	0	13	4	12	-0.1661876D-04	-0.1661260D-04	-0.6158985D-08	0.36D+10	0.000371
663	0	14	4	13	-0.1634019D-04	-0.1631246D-04	-0.2772721D-07	0.37D+10	0.001697
664	0	15	4	14	-0.1607033D-04	-0.1601546D-04	-0.5487499D-07	0.39D+10	0.003415
665	0	0	4	1	-0.2122294D-04	-0.2115743D-04	-0.6550518D-07	0.22D+10	0.003087
666	0	1	4	2	-0.2158985D-04	-0.2150770D-04	-0.8214977D-07	0.21D+10	0.003805
667	0	2	4	3	-0.2196054D-04	-0.2186155D-04	-0.9899133D-07	0.21D+10	0.004508
668	0	3	4	4	-0.2233482D-04	-0.2221903D-04	-0.1157942D-06	0.20D+10	0.005184
669	0	4	4	5	-0.2271251D-04	-0.2258014D-04	-0.1323672D-06	0.19D+10	0.005828
670	0	5	4	6	-0.2309344D-04	-0.2294497D-04	-0.1484741D-06	0.19D+10	0.006429
671	0	6	4	7	-0.2347746D-04	-0.2331351D-04	-0.1639519D-06	0.18D+10	0.006983
672	0	7	4	8	-0.2386442D-04	-0.2368581D-04	-0.1786104D-06	0.18D+10	0.007484
673	0	8	4	9	-0.2425418D-04	-0.2406191D-04	-0.1922689D-06	0.17D+10	0.007927
674	0	9	4	10	-0.2464660D-04	-0.2444186D-04	-0.2047424D-06	0.16D+10	0.008307
675	0	10	4	11	-0.2504156D-04	-0.2482568D-04	-0.2158846D-06	0.16D+10	0.008621
676	0	11	4	12	-0.2543894D-04	-0.2521341D-04	-0.2255254D-06	0.15D+10	0.008865
677	0	12	4	13	-0.2583863D-04	-0.2560512D-04	-0.2335100D-06	0.15D+10	0.009037
678	0	13	4	14	-0.2624052D-04	-0.2600082D-04	-0.2396999D-06	0.15D+10	0.009135
679	0	14	4	15	-0.2664452D-04	-0.2640057D-04	-0.2439548D-06	0.14D+10	0.009156
680	0	15	4	16	-0.2705052D-04	-0.2680440D-04	-0.2461221D-06	0.14D+10	0.009099
681	0	1	4	0	-0.2060677D-04	-0.2046752D-04	-0.1392471D-06	0.24D+10	0.006757
682	0	2	4	1	-0.1937000D-04	-0.2012781D-04	0.7578117D-06	0.27D+10	-0.039123
683	0	3	4	2	-0.1909249D-04	-0.1979157D-04	0.6990780D-06	0.27D+10	-0.036615

684	0	4	4	3	-0.1957044D-04	-0.1945873D-04	-0.1117100D-06	0.26D+10	0.005708
685	0	5	4	4	-0.1931024D-04	-0.1912930D-04	-0.1809364D-06	0.27D+10	0.009370
686	0	6	4	5	-0.1895609D-04	-0.1880323D-04	-0.1528555D-06	0.28D+10	0.008064
687	0	7	4	6	-0.1864058D-04	-0.1848050D-04	-0.1600823D-06	0.29D+10	0.008588
688	0	8	4	7	-0.1830032D-04	-0.1816107D-04	-0.1392491D-06	0.30D+10	0.007609
689	0	9	4	8	-0.1800668D-04	-0.1784493D-04	-0.1617512D-06	0.31D+10	0.008983
690	0	10	4	9	-0.1768213D-04	-0.1753203D-04	-0.1500998D-06	0.32D+10	0.008489
691	0	11	4	10	-0.1736283D-04	-0.1722236D-04	-0.1404689D-06	0.33D+10	0.008090
692	0	12	4	11	-0.1706199D-04	-0.1691590D-04	-0.1460943D-06	0.34D+10	0.008563
693	0	13	4	12	-0.1671768D-04	-0.1661260D-04	-0.1050790D-06	0.36D+10	0.006286
694	0	14	4	13	-0.1644503D-04	-0.1631246D-04	-0.1325672D-06	0.37D+10	0.008061
695	0	15	4	14	-0.1616942D-04	-0.1601546D-04	-0.1539650D-06	0.38D+10	0.009522
696	0	16	4	15	-0.1576659D-04	-0.1572155D-04	-0.4504003D-07	0.40D+10	0.002857
697	0	17	4	16	-0.1556982D-04	-0.1543072D-04	-0.1391019D-06	0.41D+10	0.008934
698	0	18	4	17	-0.1522115D-04	-0.1514295D-04	-0.7820194D-07	0.43D+10	0.005138
699	0	19	4	18	-0.1492874D-04	-0.1485821D-04	-0.7053168D-07	0.45D+10	0.004725
700	0	20	4	19	-0.1471234D-04	-0.1457651D-04	-0.1358349D-06	0.46D+10	0.009233
701	0	21	4	20	-0.1430185D-04	-0.1429778D-04	-0.4074740D-08	0.49D+10	0.000285
702	0	22	4	21	-0.1412418D-04	-0.1402202D-04	-0.1021571D-06	0.50D+10	0.007233
703	0	23	4	22	-0.1377741D-04	-0.1374923D-04	-0.2818324D-07	0.53D+10	0.002046
704	0	24	4	23	-0.1361597D-04	-0.1347938D-04	-0.1365946D-06	0.54D+10	0.010032
705	0	25	4	24	-0.1412872D-04	-0.1321245D-04	-0.9162724D-06	0.50D+10	0.064852
706	0	26	4	25	-0.1294947D-04	-0.1294841D-04	-0.1064760D-08	0.60D+10	0.000082
707	0	27	4	26	-0.1277561D-04	-0.1268725D-04	-0.8836259D-07	0.61D+10	0.006917
708	0	28	4	27	-0.1160909D-04	-0.1242897D-04	0.8198789D-06	0.00D+00	-0.070624
709	0	30	4	29	-0.1270246D-04	-0.1192093D-04	-0.7815267D-06	0.62D+10	0.061526
710	0	0	4	1	-0.2130005D-04	-0.2115743D-04	-0.1426152D-06	0.22D+10	0.006696
711	0	1	4	2	-0.2164221D-04	-0.2150770D-04	-0.1345098D-06	0.21D+10	0.006215
712	0	2	4	3	-0.2202117D-04	-0.2186155D-04	-0.1596213D-06	0.21D+10	0.007249
713	0	3	4	4	-0.2235993D-04	-0.2221903D-04	-0.1409042D-06	0.20D+10	0.006302
714	0	4	4	5	-0.2275140D-04	-0.2258014D-04	-0.1712572D-06	0.19D+10	0.007527
715	0	5	4	6	-0.2314500D-04	-0.2294497D-04	-0.2000341D-06	0.19D+10	0.008643
716	0	6	4	7	-0.2355371D-04	-0.2331351D-04	-0.2402019D-06	0.18D+10	0.010198
717	0	7	4	8	-0.2398555D-04	-0.2368581D-04	-0.2997404D-06	0.17D+10	0.012497
718	0	8	4	9	-0.2432316D-04	-0.2406191D-04	-0.2612489D-06	0.17D+10	0.010741
719	0	9	4	10	-0.2472012D-04	-0.2444186D-04	-0.2782624D-06	0.16D+10	0.011257
720	0	10	4	11	-0.2508614D-04	-0.2482568D-04	-0.2604646D-06	0.16D+10	0.010383
721	0	11	4	12	-0.2549152D-04	-0.2521341D-04	-0.2781054D-06	0.15D+10	0.010910
722	0	12	4	13	-0.2587571D-04	-0.2560512D-04	-0.2705900D-06	0.15D+10	0.010457
723	0	13	4	14	-0.2628162D-04	-0.2600082D-04	-0.2807999D-06	0.14D+10	0.010684
724	0	14	4	15	-0.2660902D-04	-0.2640057D-04	-0.2084548D-06	0.14D+10	0.007834
725	0	15	4	16	-0.2710068D-04	-0.2680440D-04	-0.2962821D-06	0.14D+10	0.010933
726	0	16	4	17	-0.2745940D-04	-0.2721236D-04	-0.2470363D-06	0.13D+10	0.008996
727	0	17	4	18	-0.2793565D-04	-0.2762450D-04	-0.3111509D-06	0.13D+10	0.011138
728	0	18	4	19	-0.2822068D-04	-0.2804086D-04	-0.1798228D-06	0.13D+10	0.006372
729	0	19	4	20	-0.2864732D-04	-0.2846148D-04	-0.1858420D-06	0.12D+10	0.006487
730	0	20	4	21	-0.2920333D-04	-0.2888643D-04	-0.3169008D-06	0.12D+10	0.010852
731	0	21	4	22	-0.2954841D-04	-0.2931572D-04	-0.2326864D-06	0.11D+10	0.007875
732	0	22	4	23	-0.3003618D-04	-0.2974944D-04	-0.2867414D-06	0.11D+10	0.009547
733	0	23	4	24	-0.3067283D-04	-0.3018762D-04	-0.4852136D-06	0.11D+10	0.015819
734	0	24	4	25	-0.2970758D-04	-0.3063031D-04	0.9227314D-06	0.11D+10	-0.031060
735	0	25	4	26	-0.3295877D-04	-0.3107758D-04	-0.1881194D-05	0.92D+09	0.057077
736	0	26	4	27	-0.3143129D-04	-0.3152945D-04	0.9815563D-07	0.10D+10	-0.003123
737	0	27	4	28	-0.3804343D-04	-0.3198599D-04	-0.6057441D-05	0.00D+00	0.159224
738	0	28	4	29	-0.3240730D-04	-0.3244727D-04	0.3997487D-07	0.95D+09	-0.001234
739	0	29	4	30	-0.3394792D-04	-0.3291333D-04	-0.1034590D-05	0.87D+09	0.030476
740	0	30	4	31	-0.3331190D-04	-0.3338423D-04	0.7233472D-07	0.90D+09	-0.002171
741	0	31	4	32	-0.3090473D-04	-0.3386003D-04	0.2955305D-05	0.00D+00	-0.095626
742	0	1	4	0	-0.2052310D-04	-0.2046752D-04	-0.5557712D-07	0.24D+10	0.002708
743	0	2	4	1	-0.2019417D-04	-0.2012781D-04	-0.6635826D-07	0.25D+10	0.003286
744	0	3	4	2	-0.1935249D-04	-0.1979157D-04	0.4390780D-06	0.27D+10	-0.022688
745	0	4	4	3	-0.1951744D-04	-0.1945873D-04	-0.5870999D-07	0.26D+10	0.003008
746	0	5	4	4	-0.1917761D-04	-0.1912930D-04	-0.4830645D-07	0.27D+10	0.002519
747	0	6	4	5	-0.1882784D-04	-0.1880323D-04	-0.2460552D-07	0.28D+10	0.001307
748	0	7	4	6	-0.1852547D-04	-0.1848050D-04	-0.4497227D-07	0.29D+10	0.002428
749	0	8	4	7	-0.1819719D-04	-0.1816107D-04	-0.3611912D-07	0.30D+10	0.001985
750	0	9	4	8	-0.1788667D-04	-0.1784493D-04	-0.4174115D-07	0.31D+10	0.002334
751	0	10	4	9	-0.1756817D-04	-0.1753203D-04	-0.3613985D-07	0.32D+10	0.002057
752	0	11	4	10	-0.1725532D-04	-0.1722236D-04	-0.3295891D-07	0.34D+10	0.001910
753	0	12	4	11	-0.1694679D-04	-0.1691590D-04	-0.3089429D-07	0.35D+10	0.001823
754	0	13	4	12	-0.1664107D-04	-0.1661260D-04	-0.2846898D-07	0.36D+10	0.001711

755	0	14	4	13	-0.1633776D-04	-0.1631246D-04	-0.2529721D-07	0.37D+10	0.001548
756	0	15	4	14	-0.1603698D-04	-0.1601546D-04	-0.2152499D-07	0.39D+10	0.001342
757	0	16	4	15	-0.1573544D-04	-0.1572155D-04	-0.1389003D-07	0.40D+10	0.000883
758	0	17	4	16	-0.1544231D-04	-0.1543072D-04	-0.1159187D-07	0.42D+10	0.000751
759	0	18	4	17	-0.1511857D-04	-0.1514295D-04	0.2437806D-07	0.44D+10	-0.001612
760	0	19	4	18	-0.1486830D-04	-0.1485821D-04	-0.1009168D-07	0.45D+10	0.000679
761	0	20	4	19	-0.1455918D-04	-0.1457651D-04	0.1732514D-07	0.47D+10	-0.001190
762	0	21	4	20	-0.1429394D-04	-0.1429778D-04	0.3835260D-08	0.49D+10	-0.000268
763	0	22	4	21	-0.1400896D-04	-0.1402202D-04	0.1306290D-07	0.51D+10	-0.000932
764	0	23	4	22	-0.1372332D-04	-0.1374923D-04	0.2590676D-07	0.53D+10	-0.001888
765	0	24	4	23	-0.1342283D-04	-0.1347938D-04	0.5654543D-07	0.56D+10	-0.004213
766	0	25	4	24	-0.1319243D-04	-0.1321245D-04	0.2001760D-07	0.57D+10	-0.001517
767	0	26	4	25	-0.1291958D-04	-0.1294841D-04	0.2882524D-07	0.60D+10	-0.002231
768	0	27	4	26	-0.1259859D-04	-0.1268725D-04	0.8865741D-07	0.63D+10	-0.007037
769	0	28	4	27	-0.1230462D-04	-0.1242897D-04	0.1243489D-06	0.66D+10	-0.010106
770	0	29	4	28	-0.1105200D-04	-0.1217353D-04	0.1121531D-05	0.00D+00	-0.101478
771	0	30	4	29	-0.1181166D-04	-0.1192093D-04	0.1092733D-06	0.72D+10	-0.009251
772	0	31	4	30	-0.1155001D-04	-0.1167115D-04	0.1211433D-06	0.75D+10	-0.010489
773	0	32	4	31	-0.1092704D-04	-0.1142418D-04	0.4971416D-06	0.84D+10	-0.045496
774	0	33	4	32	-0.1107237D-04	-0.1118000D-04	0.1076301D-06	0.82D+10	-0.009721
775	0	34	4	33	-0.1036017D-04	-0.1093859D-04	0.5784238D-06	0.93D+10	-0.055832
776	0	0	4	1	-0.2121946D-04	-0.2115743D-04	-0.6202518D-07	0.22D+10	0.002923
777	0	1	4	2	-0.2156825D-04	-0.2150770D-04	-0.6054977D-07	0.21D+10	0.002807
778	0	2	4	3	-0.2192355D-04	-0.2186155D-04	-0.6200133D-07	0.21D+10	0.002828
779	0	3	4	4	-0.2228086D-04	-0.2221903D-04	-0.6183423D-07	0.20D+10	0.002775
780	0	4	4	5	-0.2264328D-04	-0.2258014D-04	-0.6313721D-07	0.20D+10	0.002788
781	0	5	4	6	-0.2300815D-04	-0.2294497D-04	-0.6318414D-07	0.19D+10	0.002746
782	0	6	4	7	-0.2345477D-04	-0.2331351D-04	-0.1412619D-06	0.18D+10	0.006023
783	0	7	4	8	-0.2374772D-04	-0.2368581D-04	-0.6191039D-07	0.18D+10	0.002607
784	0	8	4	9	-0.2412205D-04	-0.2406191D-04	-0.6013890D-07	0.17D+10	0.002493
785	0	9	4	10	-0.2449865D-04	-0.2444186D-04	-0.5679237D-07	0.17D+10	0.002318
786	0	10	4	11	-0.2488238D-04	-0.2482568D-04	-0.5670465D-07	0.16D+10	0.002279
787	0	11	4	12	-0.2526643D-04	-0.2521341D-04	-0.5301538D-07	0.16D+10	0.002098
788	0	12	4	13	-0.2565466D-04	-0.2560512D-04	-0.4954002D-07	0.15D+10	0.001931
789	0	13	4	14	-0.2604725D-04	-0.2600082D-04	-0.4642985D-07	0.15D+10	0.001783
790	0	14	4	15	-0.2645193D-04	-0.2640057D-04	-0.5136484D-07	0.14D+10	0.001942
791	0	15	4	16	-0.2684232D-04	-0.2680440D-04	-0.3792211D-07	0.14D+10	0.001413
792	0	16	4	17	-0.2723968D-04	-0.2721236D-04	-0.2731630D-07	0.13D+10	0.001003
793	0	17	4	18	-0.2763749D-04	-0.2762450D-04	-0.1299093D-07	0.13D+10	0.000470
794	0	18	4	19	-0.2804967D-04	-0.2804086D-04	-0.8812849D-08	0.13D+10	0.000314
795	0	19	4	20	-0.2846043D-04	-0.2846148D-04	0.1048013D-08	0.12D+10	-0.000037
796	0	20	4	21	-0.2889541D-04	-0.2888643D-04	-0.8980797D-08	0.12D+10	0.000311
797	0	21	4	22	-0.2931363D-04	-0.2931572D-04	0.2093567D-08	0.12D+10	-0.000071
798	0	22	4	23	-0.2973415D-04	-0.2974944D-04	0.1528861D-07	0.11D+10	-0.000514
799	0	23	4	24	-0.3011581D-04	-0.3018762D-04	0.7180643D-07	0.11D+10	-0.002384
800	0	24	4	25	-0.3054859D-04	-0.3063031D-04	0.8172141D-07	0.11D+10	-0.002675
801	0	25	4	26	-0.3104327D-04	-0.3107758D-04	0.3430574D-07	0.10D+10	-0.001105
802	0	26	4	27	-0.3142019D-04	-0.3152945D-04	0.1092556D-06	0.10D+10	-0.003477
803	0	27	4	28	-0.3199071D-04	-0.3198599D-04	-0.4720648D-08	0.98D+09	0.000148
804	0	28	4	29	-0.3220454D-04	-0.3244727D-04	0.2427349D-06	0.96D+09	-0.007537
805	0	29	4	30	-0.3269099D-04	-0.3291333D-04	0.2223405D-06	0.94D+09	-0.006801
806	0	30	4	31	-0.3296486D-04	-0.3338423D-04	0.4193747D-06	0.92D+09	-0.012722
807	0	31	4	32	-0.3318626D-04	-0.3386003D-04	0.6737750D-06	0.91D+09	-0.020303
808	0	32	4	33	-0.3345718D-04	-0.3434079D-04	0.8836084D-06	0.89D+09	-0.026410
809	0	33	4	34	-0.3157093D-04	-0.3482656D-04	0.3255631D-05	0.00D+00	-0.103121
810	0	37	4	38	-0.3738445D-04	-0.3682107D-04	-0.5633844D-06	0.72D+09	0.015070
811	0	38	4	39	-0.3722890D-04	-0.3733288D-04	0.1039783D-06	0.72D+09	-0.002793
812	0	3	5	2	0.1150691D-05	0.1202701D-05	-0.5200975D-07	0.00D+00	-0.045199
813	0	4	5	3	0.1038703D-05	0.1252203D-05	-0.2134999D-06	0.00D+00	-0.205545
814	0	5	5	4	0.1145178D-05	0.1301292D-05	-0.1561137D-06	0.00D+00	-0.136323
815	0	6	5	5	0.1414132D-05	0.1349998D-05	0.6413369D-07	0.00D+00	0.045352
816	0	7	5	6	0.1209681D-05	0.1398300D-05	-0.1886192D-06	0.00D+00	-0.155925
817	0	8	5	7	0.1296102D-05	0.1446221D-05	-0.1501187D-06	0.00D+00	-0.115823
818	0	9	5	8	0.1255662D-05	0.1493766D-05	-0.2381044D-06	0.00D+00	-0.189625
819	0	10	5	9	0.1538866D-05	0.1540941D-05	-0.2075439D-08	0.00D+00	-0.001349
820	0	11	5	10	0.1832674D-05	0.1587753D-05	0.2449209D-06	0.00D+00	0.133641
821	0	12	5	11	0.1844361D-05	0.1634194D-05	0.2101669D-06	0.00D+00	0.113951
822	0	13	5	12	0.1899747D-05	0.1680280D-05	0.2194671D-06	0.00D+00	0.115524
823	0	14	5	13	0.2180343D-05	0.1726023D-05	0.4543199D-06	0.00D+00	0.208371
824	0	15	5	14	0.1673715D-05	0.1771407D-05	-0.9769195D-07	0.00D+00	-0.058368
825	0	16	5	15	0.1901017D-05	0.1816448D-05	0.8456857D-07	0.00D+00	0.044486

826	0	17	5	16	0.2091106D-05	0.1861166D-05	0.2299398D-06	0.00D+00	0.109961
827	0	18	5	17	0.1651002D-05	0.1905551D-05	-0.2545490D-06	0.00D+00	-0.154178
828	0	1	6	0	0.6781615D-06	0.7353987D-06	-0.5723720D-07	0.00D+00	-0.084401
829	0	2	6	1	0.7437054D-06	0.7434616D-06	0.2438237D-09	0.00D+00	0.000328
830	0	3	6	2	0.7287935D-06	0.7514650D-06	-0.2267155D-07	0.00D+00	-0.031108
831	0	4	6	3	0.7158213D-06	0.7594392D-06	-0.4361789D-07	0.00D+00	-0.060934
832	0	5	6	4	0.7147719D-06	0.7673774D-06	-0.5260550D-07	0.00D+00	-0.073598
833	0	6	6	5	0.7469475D-06	0.7752630D-06	-0.2831552D-07	0.00D+00	-0.037908
834	0	7	6	6	0.7466217D-06	0.7831140D-06	-0.3649228D-07	0.00D+00	-0.048877
835	0	8	6	7	0.7475971D-06	0.7909329D-06	-0.4333577D-07	0.00D+00	-0.057967
836	0	9	6	8	0.7614985D-06	0.7987025D-06	-0.3720404D-07	0.00D+00	-0.048856
837	0	10	6	9	0.7828626D-06	0.8064416D-06	-0.2357903D-07	0.00D+00	-0.030119
838	0	11	6	10	0.7832799D-06	0.8141505D-06	-0.3087056D-07	0.00D+00	-0.039412
839	0	12	6	11	0.7885145D-06	0.8218227D-06	-0.3330821D-07	0.00D+00	-0.042242
840	0	13	6	12	0.7952784D-06	0.8294690D-06	-0.3419058D-07	0.00D+00	-0.042992
841	0	14	6	13	0.8125301D-06	0.8370747D-06	-0.2454464D-07	0.00D+00	-0.030208
842	0	15	6	14	0.8321974D-06	0.8446491D-06	-0.1245173D-07	0.00D+00	-0.014962
843	0	16	6	15	0.8375674D-06	0.8522095D-06	-0.1464213D-07	0.00D+00	-0.017482
844	0	17	6	16	0.8323692D-06	0.8597387D-06	-0.2736950D-07	0.00D+00	-0.032881
845	0	18	6	17	0.8218948D-06	0.8672282D-06	-0.4533337D-07	0.00D+00	-0.055157
846	0	19	6	18	0.7987821D-06	0.8747043D-06	-0.7592222D-07	0.00D+00	-0.095047
847	0	20	6	19	0.8705461D-06	0.8821464D-06	-0.1160034D-07	0.00D+00	-0.013325
848	0	0	6	1	0.6637963D-06	0.7191280D-06	-0.5533173D-07	0.00D+00	-0.083356
849	0	1	6	2	0.7287644D-06	0.7109316D-06	0.1783279D-07	0.00D+00	0.024470
850	0	2	6	3	0.6814027D-06	0.7026843D-06	-0.2128163D-07	0.00D+00	-0.031232
851	0	3	6	4	0.6519976D-06	0.6943708D-06	-0.4237320D-07	0.00D+00	-0.064990
852	0	4	6	5	0.6676359D-06	0.6860147D-06	-0.1837878D-07	0.00D+00	-0.027528
853	0	5	6	6	0.6542812D-06	0.6776126D-06	-0.2333143D-07	0.00D+00	-0.035660
854	0	6	6	7	0.6148423D-06	0.6691460D-06	-0.5430366D-07	0.00D+00	-0.088321
855	0	7	6	8	0.6274726D-06	0.6606233D-06	-0.3315066D-07	0.00D+00	-0.052832
856	0	8	6	9	0.6258880D-06	0.6520454D-06	-0.2615737D-07	0.00D+00	-0.041792
857	0	9	6	10	0.6042367D-06	0.6433990D-06	-0.3916230D-07	0.00D+00	-0.064813
858	0	10	6	11	0.6049619D-06	0.6346984D-06	-0.2973654D-07	0.00D+00	-0.049154
859	0	11	6	12	0.5872681D-06	0.6259403D-06	-0.3867224D-07	0.00D+00	-0.065851
860	1	14	4	13	-0.7826485D-03	-0.7978551D-03	0.1520657D-04	0.16D+07	-0.019430
861	1	15	4	14	-0.7886742D-03	-0.7936689D-03	0.4994674D-05	0.16D+07	-0.006333
862	1	1	4	0	-0.8747712D-03	-0.8570153D-03	-0.1775592D-04	0.13D+07	0.020298
863	1	2	4	1	-0.8300564D-03	-0.8521463D-03	0.2208989D-04	0.15D+07	-0.026613
864	1	3	4	2	-0.8538633D-03	-0.8473312D-03	-0.6532086D-05	0.14D+07	0.007650
865	1	4	4	3	-0.8631158D-03	-0.8425698D-03	-0.2054600D-04	0.13D+07	0.023804
866	1	5	4	4	-0.1063504D-02	-0.8378618D-03	-0.2256422D-03	0.00D+00	0.212169
867	1	6	4	5	-0.8464347D-03	-0.8332069D-03	-0.1322778D-04	0.14D+07	0.015628
868	1	7	4	6	-0.8384119D-03	-0.8286050D-03	-0.9806895D-05	0.14D+07	0.011697
869	1	8	4	7	-0.8568882D-03	-0.8240558D-03	-0.3283243D-04	0.14D+07	0.038316
870	1	9	4	8	-0.8386739D-03	-0.8195590D-03	-0.1911488D-04	0.14D+07	0.022792
871	1	10	4	9	-0.8459599D-03	-0.8151145D-03	-0.3084537D-04	0.14D+07	0.036462
872	1	11	4	10	-0.8092902D-03	-0.8107221D-03	0.1431892D-05	0.15D+07	-0.001769
873	1	12	4	11	-0.8098315D-03	-0.8063815D-03	-0.3450003D-05	0.15D+07	0.004260
874	1	13	4	12	-0.8157900D-03	-0.8020925D-03	-0.1369745D-04	0.15D+07	0.016790
875	1	14	4	13	-0.8248629D-03	-0.7978551D-03	-0.2700783D-04	0.15D+07	0.032742
876	1	15	4	14	-0.8045300D-03	-0.7936689D-03	-0.1086113D-04	0.15D+07	0.013500
877	1	16	4	15	-0.7995977D-03	-0.7895338D-03	-0.1006394D-04	0.16D+07	0.012586
878	1	17	4	16	-0.7694562D-03	-0.7854496D-03	0.1599338D-04	0.17D+07	-0.020785
879	1	18	4	17	-0.7699712D-03	-0.7814162D-03	0.1144498D-04	0.17D+07	-0.014864
880	1	19	4	18	-0.7895303D-03	-0.7774334D-03	-0.1209693D-04	0.16D+07	0.015322
881	1	20	4	19	-0.8423465D-03	-0.7735010D-03	-0.6884550D-04	0.14D+07	0.081731
882	1	21	4	20	-0.7651987D-03	-0.7696189D-03	0.4420245D-05	0.17D+07	-0.005777
883	1	22	4	21	-0.8372380D-03	-0.7657870D-03	-0.7145095D-04	0.14D+07	0.085341
884	1	23	4	22	-0.6738823D-03	-0.7620052D-03	0.8812288D-04	0.00D+00	-0.130769
885	1	24	4	23	-0.8309432D-03	-0.7582732D-03	-0.7266999D-04	0.14D+07	0.087455
886	1	25	4	24	-0.8003815D-03	-0.7545910D-03	-0.4579046D-04	0.16D+07	0.057211
887	1	0	4	1	-0.8300135D-03	-0.8669160D-03	0.3690250D-04	0.15D+07	-0.044460
888	1	1	4	2	-0.8715563D-03	-0.8719483D-03	0.3920066D-06	0.13D+07	-0.000450
889	1	2	4	3	-0.9268275D-03	-0.8770356D-03	-0.4979192D-04	0.12D+07	0.053723
890	1	3	4	4	-0.9166089D-03	-0.8821782D-03	-0.3443074D-04	0.12D+07	0.037563
891	1	4	4	5	-0.8928153D-03	-0.8873763D-03	-0.5438982D-05	0.13D+07	0.006092
892	1	5	4	6	-0.9022312D-03	-0.8926303D-03	-0.9600856D-05	0.12D+07	0.010641
893	1	6	4	7	-0.8827989D-03	-0.8979406D-03	0.1514166D-04	0.13D+07	-0.017152
894	1	7	4	8	-0.9117731D-03	-0.9033073D-03	-0.8465799D-05	0.12D+07	0.009285
895	1	8	4	9	-0.9173332D-03	-0.9087309D-03	-0.8602343D-05	0.12D+07	0.009378
896	1	9	4	10	-0.9337497D-03	-0.9142116D-03	-0.1953813D-04	0.11D+07	0.020924

897	1	10	4	11	-0.9418192D-03	-0.9197498D-03	-0.2206941D-04	0.11D+07	0.023433
898	1	11	4	12	-0.9331414D-03	-0.9253459D-03	-0.7795538D-05	0.11D+07	0.008354
899	1	12	4	13	-0.9363112D-03	-0.9310001D-03	-0.5311077D-05	0.11D+07	0.005672
900	1	13	4	14	-0.9551414D-03	-0.9367129D-03	-0.1842846D-04	0.11D+07	0.019294
901	1	14	4	15	-0.1022114D-02	-0.9424847D-03	-0.7962931D-04	0.96D+06	0.077906
902	1	15	4	16	-0.9535475D-03	-0.9483157D-03	-0.5231769D-05	0.11D+07	0.005487
903	1	16	4	17	-0.9194256D-03	-0.9542065D-03	0.3478086D-04	0.12D+07	-0.037829
904	1	17	4	18	-0.9520251D-03	-0.9601573D-03	0.8132158D-05	0.11D+07	-0.008542
905	1	18	4	19	-0.1072700D-02	-0.9661685D-03	-0.1065315D-03	0.00D+00	0.099312
906	1	20	4	21	-0.9228706D-03	-0.9783741D-03	0.5550353D-04	0.12D+07	-0.060142
907	1	6	5	5	-0.4533281D-04	-0.4614686D-04	0.8140519D-06	0.49D+09	-0.017957
908	1	11	5	10	-0.4230125D-04	-0.4258458D-04	0.2833282D-06	0.56D+09	-0.006698
909	1	12	5	11	-0.4661456D-04	-0.4189450D-04	-0.4720062D-05	0.00D+00	0.101257
910	1	15	5	14	-0.3716700D-04	-0.3986796D-04	0.2700958D-05	0.72D+09	-0.072671
911	1	2	5	1	-0.4915838D-04	-0.4913464D-04	-0.2374242D-07	0.41D+09	0.000483
912	1	3	5	2	-0.4725102D-04	-0.4837593D-04	0.1124914D-05	0.45D+09	-0.023807
913	1	4	5	3	-0.4567179D-04	-0.4762512D-04	0.1953329D-05	0.48D+09	-0.042769
914	1	6	5	5	-0.4638212D-04	-0.4614686D-04	-0.2352581D-06	0.46D+09	0.005072
915	1	7	5	6	-0.4401777D-04	-0.4541929D-04	0.1401516D-05	0.52D+09	-0.031840
916	1	9	5	8	-0.4303643D-04	-0.4398694D-04	0.9505145D-06	0.54D+09	-0.022086
917	1	10	5	9	-0.4300715D-04	-0.4328204D-04	0.2748905D-06	0.54D+09	-0.006392
918	1	11	5	10	-0.4230461D-04	-0.4258458D-04	0.2799682D-06	0.56D+09	-0.006618
919	1	12	5	11	-0.4754303D-04	-0.4189450D-04	-0.5648532D-05	0.00D+00	0.118809
920	1	13	5	12	-0.4266778D-04	-0.4121174D-04	-0.1456040D-05	0.55D+09	0.034125
921	1	14	5	13	-0.3923897D-04	-0.4053624D-04	0.1297273D-05	0.65D+09	-0.033061
922	1	15	5	14	-0.4295798D-04	-0.3986796D-04	-0.3090022D-05	0.54D+09	0.071931
923	1	16	5	15	-0.3807797D-04	-0.3920683D-04	0.1128865D-05	0.69D+09	-0.029646
924	1	17	5	16	-0.3653882D-04	-0.3855282D-04	0.2014002D-05	0.75D+09	-0.055120
925	1	18	5	17	-0.3766857D-04	-0.3790586D-04	0.2372916D-06	0.70D+09	-0.006299
926	1	0	5	1	-0.5685885D-04	-0.5145873D-04	-0.5400124D-05	0.31D+09	0.094974
927	1	9	5	10	-0.5945203D-04	-0.5888070D-04	-0.5713278D-06	0.28D+09	0.009610
928	1	10	5	11	-0.5645004D-04	-0.5974877D-04	0.3298726D-05	0.31D+09	-0.058436
929	1	11	5	12	-0.6076415D-04	-0.6062583D-04	-0.1383165D-06	0.27D+09	0.002276
930	1	12	5	13	-0.6076033D-04	-0.6151199D-04	0.7516638D-06	0.27D+09	-0.012371
931	1	13	5	14	-0.6356474D-04	-0.6240734D-04	-0.1157396D-05	0.25D+09	0.018208
932	1	14	5	15	-0.6484051D-04	-0.6331197D-04	-0.1528543D-05	0.24D+09	0.023574
933	1	15	5	16	-0.6450295D-04	-0.6422597D-04	-0.2769790D-06	0.24D+09	0.004294
934	1	17	5	18	-0.5912267D-04	-0.6608253D-04	0.6959857D-05	0.00D+00	-0.117719
935	1	18	5	19	-0.7530868D-04	-0.6702527D-04	-0.8283406D-05	0.00D+00	0.109993
936	1	19	5	20	-0.1277297D-03	-0.6797783D-04	-0.5975187D-04	0.00D+00	0.467799
937	1	20	5	21	-0.8178134D-04	-0.6894028D-04	-0.1284106D-04	0.00D+00	0.157017
938	4	10	5	9	-0.2380000D+00	-0.2394523D+00	0.1452333D-02	0.18D+02	-0.006102
939	4	12	5	11	-0.2380000D+00	-0.2394898D+00	0.1489823D-02	0.18D+02	-0.006260
940	5	9	6	8	-0.2610000D+00	-0.2618779D+00	0.8778574D-03	0.15D+02	-0.003363
941	5	11	6	10	-0.2590000D+00	-0.2619280D+00	0.2928040D-02	0.15D+02	-0.011305
942	5	13	6	12	-0.2590000D+00	-0.2619852D+00	0.2985151D-02	0.15D+02	-0.011526
943	6	9	7	8	-0.2770000D+00	-0.2823510D+00	0.5351016D-02	0.13D+02	-0.019318
944	6	10	7	9	-0.2750000D+00	-0.2823841D+00	0.7384147D-02	0.13D+02	-0.026851
945	6	13	7	12	-0.2770000D+00	-0.2824943D+00	0.5494292D-02	0.13D+02	-0.019835
946	7	8	8	7	-0.2950000D+00	-0.3011889D+00	0.6188930D-02	0.11D+02	-0.020979
947	7	9	8	8	-0.3040000D+00	-0.3012301D+00	-0.2769944D-02	0.11D+02	0.009112
948	7	12	8	11	-0.2940000D+00	-0.3013643D+00	0.7364312D-02	0.12D+02	-0.025049
949	8	11	9	10	-0.3130000D+00	-0.3188811D+00	0.5881133D-02	0.10D+02	-0.018790
950	9	12	10	11	-0.3260000D+00	-0.3353625D+00	0.9362526D-02	0.94D+01	-0.028719
951	10	10	11	9	-0.3440000D+00	-0.3506135D+00	0.6613519D-02	0.85D+01	-0.019225
952	0	30	3	29	-0.3567939D-03	-0.3551900D-03	-0.1603930D-05	0.00D+00	0.004495
953	0	27	3	26	-0.3612520D-03	-0.3604309D-03	-0.8210741D-06	0.38D+09	0.002273
954	0	27	3	26	-0.3611997D-03	-0.3604309D-03	-0.7687741D-06	0.38D+09	0.002128
955	0	23	3	24	-0.4859220D-03	-0.4850186D-03	-0.9033994D-06	0.21D+09	0.001859
956	0	26	3	27	-0.4955082D-03	-0.4946396D-03	-0.8686316D-06	0.20D+09	0.001753
957	0	27	3	28	-0.4985821D-03	-0.4979099D-03	-0.6722016D-06	0.20D+09	0.001348
958	0	28	3	29	-0.5025066D-03	-0.5012123D-03	-0.1294300D-05	0.20D+09	0.002576
959	0	29	3	30	-0.5051393D-03	-0.5045470D-03	-0.5922898D-06	0.20D+09	0.001173
960	0	30	3	29	-0.3564310D-03	-0.3551900D-03	-0.1241030D-05	0.39D+09	0.003482
961	0	27	3	26	-0.3610770D-03	-0.3604309D-03	-0.6460741D-06	0.38D+09	0.001789
962	0	23	3	24	-0.4860172D-03	-0.4850186D-03	-0.9985994D-06	0.21D+09	0.002055
963	0	23	3	24	-0.4858382D-03	-0.4850186D-03	-0.8195994D-06	0.21D+09	0.001687
964	0	26	3	27	-0.4955806D-03	-0.4946396D-03	-0.9410316D-06	0.20D+09	0.001899
965	0	27	3	28	-0.4988299D-03	-0.4979099D-03	-0.9200016D-06	0.20D+09	0.001844
966	0	28	3	29	-0.5022105D-03	-0.5012123D-03	-0.9982004D-06	0.20D+09	0.001988
967	0	29	3	30	-0.5054508D-03	-0.5045470D-03	-0.9037898D-06	0.20D+09	0.001788

968	0	20	3	19	-0.3747834D-03	-0.3735135D-03	-0.1269909D-05	0.36D+09	0.003388
969	0	15	3	14	-0.3845968D-03	-0.3835995D-03	-0.9973312D-06	0.34D+09	0.002593
970	0	10	3	9	-0.3952733D-03	-0.3943144D-03	-0.9589238D-06	0.32D+09	0.002426
971	0	5	3	4	-0.4064519D-03	-0.4056702D-03	-0.7816775D-06	0.30D+09	0.001923
972	0	5	3	6	-0.4337629D-03	-0.4329811D-03	-0.7817607D-06	0.27D+09	0.001802
973	0	10	3	11	-0.4473270D-03	-0.4464921D-03	-0.8349301D-06	0.25D+09	0.001866
974	0	15	3	16	-0.4615291D-03	-0.4607166D-03	-0.8124682D-06	0.23D+09	0.001760
975	0	20	3	21	-0.4762063D-03	-0.4756778D-03	-0.5284764D-06	0.22D+09	0.001110
976	0	1	7	0	0.2060000D-06	0.2162148D-06	-0.1021483D-07	0.00D+00	-0.049587
977	0	2	7	1	0.2070000D-06	0.2176220D-06	-0.1062199D-07	0.00D+00	-0.051314
978	0	3	7	2	0.2090000D-06	0.2190318D-06	-0.1003183D-07	0.00D+00	-0.047999
979	0	4	7	3	0.2100000D-06	0.2204284D-06	-0.1042836D-07	0.00D+00	-0.049659
980	0	5	7	4	0.2110000D-06	0.2218172D-06	-0.1081720D-07	0.00D+00	-0.051266
981	0	6	7	5	0.2130000D-06	0.2232119D-06	-0.1021188D-07	0.00D+00	-0.047943
982	0	7	7	6	0.2140000D-06	0.2246024D-06	-0.1060236D-07	0.00D+00	-0.049544
983	0	8	7	7	0.2160000D-06	0.2259879D-06	-0.9987936D-08	0.00D+00	-0.046240
984	0	9	7	8	0.2170000D-06	0.2273632D-06	-0.1036317D-07	0.00D+00	-0.047757
985	0	10	7	9	0.2180000D-06	0.2287537D-06	-0.1075371D-07	0.00D+00	-0.049329
986	0	11	7	10	0.2200000D-06	0.2301282D-06	-0.1012821D-07	0.00D+00	-0.046037
987	0	12	7	11	0.2210000D-06	0.2315119D-06	-0.1051189D-07	0.00D+00	-0.047565
988	0	13	7	12	0.2230000D-06	0.2328830D-06	-0.9882983D-08	0.00D+00	-0.044318
989	0	14	7	13	0.2240000D-06	0.2342630D-06	-0.1026298D-07	0.00D+00	-0.045817
990	0	15	7	14	0.2250000D-06	0.2356343D-06	-0.1063428D-07	0.00D+00	-0.047263
991	0	16	7	15	0.2270000D-06	0.2370017D-06	-0.1000172D-07	0.00D+00	-0.044060
992	0	17	7	16	0.2280000D-06	0.2383717D-06	-0.1037168D-07	0.00D+00	-0.045490
993	0	18	7	17	0.2300000D-06	0.2397422D-06	-0.9742227D-08	0.00D+00	-0.042358
994	0	19	7	18	0.2310000D-06	0.2411200D-06	-0.1011998D-07	0.00D+00	-0.043809
995	0	20	7	19	0.2320000D-06	0.2424943D-06	-0.1049435D-07	0.00D+00	-0.045234
996	0	0	7	1	0.2030000D-06	0.2133919D-06	-0.1039193D-07	0.00D+00	-0.051192
997	0	1	7	2	0.2020000D-06	0.2119710D-06	-0.9970994D-08	0.00D+00	-0.049361
998	0	2	7	3	0.2000000D-06	0.2105338D-06	-0.1053377D-07	0.00D+00	-0.052669
999	0	3	7	4	0.1990000D-06	0.2091135D-06	-0.1011347D-07	0.00D+00	-0.050821
1000	0	4	7	5	0.1970000D-06	0.2076780D-06	-0.1067800D-07	0.00D+00	-0.054203
1001	0	5	7	6	0.1960000D-06	0.2062296D-06	-0.1022965D-07	0.00D+00	-0.052192
1002	0	6	7	7	0.1940000D-06	0.2047802D-06	-0.1078016D-07	0.00D+00	-0.055568
1003	0	7	7	8	0.1930000D-06	0.2033181D-06	-0.1031807D-07	0.00D+00	-0.053462
1004	0	8	7	9	0.1910000D-06	0.2018596D-06	-0.1085957D-07	0.00D+00	-0.056856
1005	0	9	7	10	0.1900000D-06	0.2003841D-06	-0.1038410D-07	0.00D+00	-0.054653
1006	0	10	7	11	0.1880000D-06	0.1989150D-06	-0.1091500D-07	0.00D+00	-0.058059
1007	0	11	7	12	0.1870000D-06	0.1974190D-06	-0.1041896D-07	0.00D+00	-0.055716
1008	0	12	7	13	0.1850000D-06	0.1959303D-06	-0.1093034D-07	0.00D+00	-0.059083
1009	0	13	7	14	0.1840000D-06	0.1944268D-06	-0.1042678D-07	0.00D+00	-0.056667
1010	0	14	7	15	0.1820000D-06	0.1929221D-06	-0.1092208D-07	0.00D+00	-0.060011
1011	0	15	7	16	0.1810000D-06	0.1914000D-06	-0.1040003D-07	0.00D+00	-0.057459
1012	0	16	7	17	0.1790000D-06	0.1898614D-06	-0.1086140D-07	0.00D+00	-0.060678
1013	0	17	7	18	0.1780000D-06	0.1883216D-06	-0.1032163D-07	0.00D+00	-0.057987
1014	0	18	7	19	0.1760000D-06	0.1867757D-06	-0.1077568D-07	0.00D+00	-0.061225
1015	0	19	7	20	0.1750000D-06	0.1852206D-06	-0.1022063D-07	0.00D+00	-0.058404

a
Δ=(<v,J|μ|v',J'>_ref - <v,J|μ|v',J'>_calc)/<v,J|μ|v',J'>_ref

Table S4. The parameters of the morphed electric dipole moment functions of CO [A.A. Balashov, K. Bielska, G. Li, A.A. Kyuberis, S. Wojtewicz, J. Domyslawska, R. Ciurylo, N.F. Zobov, D. Lisak, J. Tennyson, O.L. Polyansky, J.Chem.Phys.158,234306(2023)].

Parameter	Fit-0	Fit-a	Fit-b	Fit-c	Fit-d	Fit-d
r_e, Å	1.927546	1.9173(10)	1.91467(29)	1.91172(28)	1.91768(28)	1.92503(28)
ρ_{ij} (κ=-3), Å	1.929974	1.9522(22)	1.95411(66)	1.94402(61)	1.91187(61)	1.75477(51)
D_e, D	1.482565	1.4733(10)	1.47143(86)	1.44778(97)	1.45527(83)	1.46107(83)
α	1.0	1.0	1.0	1.004(a)	1.0	1.0
β	1.0	1.0	1.0	1.0	0.992(a)	1.0
γ	0.0	0.0	0.0	0.0	0.0	-0.05
σ(b), D	0.03162		0.01475	0.01432	0.01432	0.01431

(a) Fixed after a preliminary determination. (b) The standard deviation of the fit.

Table S5. The reduced ab initio electric dipole moment function of CO [ab initio function from S.R. Langhoff,J.C.W. Bauschlicher,J.Chem.Phys.102,5220(1995); evaluated using κ=-3].

ρ	m	ρ	m	ρ	m	ρ	m	ρ	m
0.0073406	0.8395087	0.3325772	0.0686587	1.2863336	0.8954116	2.8640754	0.0734897	4.8458452	-0.0076779
0.0077226	0.8675749	0.3356681	0.0790950	1.2927911	0.8911254	2.8732043	0.0722611	4.8563307	-0.0077354
0.0081148	0.8952023	0.3387754	0.0895076	1.2992646	0.8867675	2.8823434	0.0710499	4.8668200	-0.0077921
0.0085172	0.9223772	0.3418989	0.0998953	1.3057542	0.8823391	2.8914924	0.0698553	4.8773131	-0.0078473
0.0089299	0.9490881	0.3450388	0.1102571	1.3122598	0.8778415	2.9006515	0.0686766	4.8878100	-0.0079019
0.0093529	0.9753304	0.3481950	0.1205920	1.3187815	0.8732761	2.9098205	0.0675153	4.8983106	-0.0079555
0.0097862	1.0011015	0.3513675	0.1308989	1.3253192	0.8686442	2.9189995	0.0663699	4.9088149	-0.0080077
0.0102300	1.0264052	0.3545564	0.1411768	1.3318729	0.8639472	2.9281883	0.0652403	4.9193229	-0.0080592
0.0106841	1.0512484	0.3577617	0.1514248	1.3384426	0.8591867	2.9373870	0.0641259	4.9298346	-0.0081099
0.0111488	1.0756423	0.3609835	0.1616418	1.3450282	0.8543641	2.9465956	0.0630282	4.9403500	-0.0081590
0.0116239	1.0996028	0.3642216	0.1718268	1.3516297	0.8494808	2.9558139	0.0619448	4.9508689	-0.0082074
0.0121095	1.1231507	0.3674762	0.1819789	1.3582472	0.8445384	2.9650420	0.0608773	4.9613915	-0.0082551
0.0126057	1.1463118	0.3707472	0.1920973	1.3648806	0.8395386	2.9742798	0.0598242	4.9719176	-0.0083020
0.0131126	1.1691169	0.3740348	0.2021808	1.3715298	0.8344828	2.9835272	0.0587854	4.9824474	-0.0083475
0.0136300	1.1916017	0.3773388	0.2122286	1.3781949	0.8293727	2.9927844	0.0577619	4.9929806	-0.0083928
0.0141582	1.2138110	0.3806593	0.2222399	1.3848758	0.8242100	3.0020511	0.0567526	5.0035173	-0.0084367
0.0146970	1.2357920	0.3839963	0.2322138	1.3915725	0.8189963	3.0113274	0.0557570	5.0140575	-0.0084798
0.0152467	1.2576039	0.3873499	0.2421493	1.3982850	0.8137335	3.0206133	0.0547758	5.0246012	-0.0085214
0.0158071	1.2793106	0.3907200	0.2520457	1.4050133	0.8084232	3.0299086	0.0538082	5.0351483	-0.0085630
0.0163783	1.3009881	0.3941067	0.2619020	1.4117573	0.8030671	3.0392135	0.0528534	5.0456988	-0.0086039
0.0169604	1.3227227	0.3975100	0.2717175	1.4185170	0.7976672	3.0485277	0.0519130	5.0562527	-0.0086433
0.0175534	1.3446152	0.4009298	0.2814913	1.4252925	0.7922253	3.0578514	0.0509847	5.0668099	-0.0086818
0.0181573	1.3667786	0.4043663	0.2912226	1.4320836	0.7867431	3.0671844	0.0500701	5.0773705	-0.0087204
0.0187722	1.3893466	0.4078194	0.3009108	1.4388903	0.7812226	3.0765268	0.0491682	5.0879344	-0.0087575
0.0193982	1.4124711	0.4112892	0.3105549	1.4457127	0.7756656	3.0858785	0.0482793	5.0985016	-0.0087938
0.0200351	1.4363305	0.4147756	0.3201543	1.4525507	0.7700742	3.0952394	0.0474024	5.1090721	-0.0088293
0.0206832	1.4611291	0.4182787	0.3297081	1.4594042	0.7644503	3.1046096	0.0465377	5.1196458	-0.0088649
0.0213423	1.4871038	0.4217984	0.3392157	1.4662734	0.7587958	3.1139889	0.0456859	5.1302227	-0.0088990
0.0220127	1.5145249	0.4253349	0.3486764	1.4731580	0.7531127	3.1233775	0.0448453	5.1408029	-0.0089322
0.0226942	1.5436954	0.4288880	0.3580894	1.4800582	0.7474030	3.1327751	0.0440169	5.1513862	-0.0089648
0.0233870	1.5749418	0.4324579	0.3674540	1.4869739	0.7416688	3.1421818	0.0431999	5.1619727	-0.0089973
0.0240910	1.6085866	0.4360445	0.3767696	1.4939050	0.7359121	3.1515976	0.0423941	5.1725623	-0.0090283
0.0248063	1.6448825	0.4396479	0.3860355	1.5008515	0.7301351	3.1610224	0.0416005	5.1831550	-0.0090594
0.0255330	1.6838615	0.4432680	0.3952510	1.5078135	0.7243397	3.1704562	0.0408175	5.1937508	-0.0090889
0.0262710	1.7250059	0.4469049	0.4044156	1.5147909	0.7185281	3.1798989	0.0400451	5.2043496	-0.0091184
0.0270205	1.7665377	0.4505586	0.4135285	1.5217837	0.7127024	3.1893506	0.0392839	5.2149515	-0.0091471
0.0277814	1.8039412	0.4542291	0.4225892	1.5287917	0.7068646	3.1988111	0.0385342	5.2255564	-0.0091751
0.0285538	1.8270684	0.4579164	0.4315970	1.5358152	0.7010171	3.2082805	0.0377943	5.2361643	-0.0092023
0.0293377	1.8154393	0.4616205	0.4405514	1.5428539	0.6951619	3.2177587	0.0370650	5.2467752	-0.0092288
0.0301332	1.7344585	0.4653414	0.4494517	1.5499078	0.6893012	3.2272456	0.0363455	5.2573891	-0.0092545
0.0309402	1.5447277	0.4690792	0.4582974	1.5569771	0.6834371	3.2367413	0.0356367	5.2680058	-0.0092803
0.0317589	1.2382115	0.4728339	0.4670880	1.5640615	0.6775719	3.2462457	0.0349384	5.2786255	-0.0093052
0.0325893	0.8675036	0.4766054	0.4758228	1.5711611	0.6717076	3.2557588	0.0342491	5.2892481	-0.0093294
0.0334313	0.5089188	0.4803938	0.4845013	1.5782759	0.6658466	3.2652806	0.0335698	5.2998735	-0.0093529
0.0342851	0.2071192	0.4841990	0.4931230	1.5854059	0.6599910	3.2748109	0.0329002	5.3105018	-0.0093755
0.0351507	-0.0305021	0.4880212	0.5016874	1.5925509	0.6541430	3.2843498	0.0322398	5.3211329	-0.0093983
0.0360280	-0.2136252	0.4918602	0.5101938	1.5997111	0.6483048	3.2938972	0.0315892	5.3317668	-0.0094202
0.0369173	-0.3549536	0.4957162	0.5186419	1.6068863	0.6424786	3.3034532	0.0309476	5.3424034	-0.0094414
0.0378184	-0.4652572	0.4995892	0.5270312	1.6140766	0.6366666	3.3130176	0.0303143	5.3530429	-0.0094618
0.0387314	-0.5525987	0.5034790	0.5353611	1.6212819	0.6308711	3.3225904	0.0296910	5.3636850	-0.0094823
0.0396564	-0.6227770	0.5073858	0.5436311	1.6285022	0.6250941	3.3321716	0.0290766	5.3743299	-0.0095019
0.0405933	-0.6799329	0.5113096	0.5518407	1.6357374	0.6193380	3.3417612	0.0284707	5.3849775	-0.0095208
0.0415423	-0.7270433	0.5152503	0.5599897	1.6429875	0.6136048	3.3513591	0.0278730	5.3956277	-0.0095397
0.0425034	-0.7662760	0.5192080	0.5680773	1.6502526	0.6078968	3.3609654	0.0272836	5.4062806	-0.0095579
0.0434765	-0.7992331	0.5231826	0.5761032	1.6575326	0.6022162	3.3705799	0.0267033	5.4169361	-0.0095753
0.0444618	-0.8271169	0.5271743	0.5840669	1.6648274	0.5965650	3.3802026	0.0261306	5.4275943	-0.0095927
0.0454592	-0.8508445	0.5311829	0.5919680	1.6721370	0.5909456	3.3898335	0.0255670	5.4382550	-0.0096094
0.0464688	-0.8711247	0.5352086	0.5998060	1.6794615	0.5853599	3.3994725	0.0250109	5.4489183	-0.0096252
0.0474907	-0.8885129	0.5392513	0.6075806	1.6868007	0.5798102	3.4091197	0.0244625	5.4595841	-0.0096411
0.0485248	-0.9034496	0.5433110	0.6152912	1.6941546	0.5742986	3.4187750	0.0239223	5.4702525	-0.0096562
0.0495713	-0.9162902	0.5473877	0.6229376	1.7015233	0.5688271	3.4284383	0.0233889	5.4809234	-0.0096714
0.0506301	-0.9273216	0.5514814	0.6305191	1.7089067	0.5619795	3.4381097	0.0228639	5.4915967	-0.0096857
0.0517012	-0.9367788	0.5555922	0.6380355	1.7163047	0.5551790	3.4477891	0.0223464	5.5022726	-0.0096994
0.0527848	-0.9448581	0.5597201	0.6454863	1.7237174	0.5484268	3.4574764	0.0218365	5.5129508	-0.0097130
0.0538808	-0.9517221	0.5638650	0.6528711	1.7311447	0.5417231	3.4671716	0.0213334	5.5236315	-0.0097259
0.0549893	-0.9575071	0.5680270	0.6601895	1.7385865	0.5350692	3.4768747	0.0208378	5.5343146	-0.0097387
0.0561104	-0.9623296	0.5722060	0.6674411	1.7460429	0.5284653	3.4865857	0.0203491	5.5450001	-0.0097508
0.0572439	-0.9662887	0.5764021	0.6746257	1.7535139	0.5219129	3.4963045	0.0198672	5.5556880	-0.0097622
0.0583901	-0.9694697	0.5806153	0.6817426	1.7609993	0.5154127	3.5060311	0.0193921	5.5663782	-0.0097736
0.0595489	-0.9719452	0.5848455	0.6887915	1.7684992	0.5089646	3.5157654	0.0189245	5.5770707	-0.0097849
0.0607203	-0.9737803	0.5890929	0.6957721	1.7760135	0.5025703	3.5255075	0.0184631	5.5877655	-0.0097947
0.0619044	-0.9750297	0.5933574	0.7026840	1.7835423	0.4962289	3.5352572	0.0180084	5.5984626	-0.0098053
0.0631013	-0.9757426	0.5976389	0.7095268	1.7910854	0.4899427	3.5450146	0.0175605	5.6091620	-0.0098152
0.0643109	-0.9759622	0.6019376	0.7163001	1.7986429	0.4837103	3.5547796	0.0171187	5.6198636	-0.0098242
0.0655333	-0.9757266	0.6062534	0.7230036	1.8062147	0.4775339	3.5645523	0.0166837	5.6305675	-0.0098333
0.0667685	-0.9750689	0.6105862	0.7296367	1.8138007	0.4714127	3.5743324	0.0162547	5.6412736	-0.0098416
0.0680166	-0.9740199	0.6149362	0.7361993	1.8214011	0.4653474	3.5841201	0.0158318	5.6519818	-0.0098499
0.0692776	-0.9726061	0.6193034	0.7426909	1.8290157	0.4593382	3.5939153	0.0154150	5.6626922	-0.0098582
0.0705515	-0.9708522	0.6236876	0.7491111	1.8366445	0.4533857	3.6037179	0.0150041	5.6734048	-0.0098658
0.0718384	-0.9687795	0.6280890	0.7554596	1.8442875	0.4474899	3.6135279	0.0145994	5.6841195	-0.0098734
0.0731382	-0.9664078	0.6325076	0.7617360	1.8519446	0.4416501	3.6233454	0.0141999	5.6948363	-0.0098802
0.0744511	-0.9637547	0.6369432	0.7679400	1.8596158	0.4358686	3.6331702	0.0138073	5.7055553	-0.0098863
0.0757771	-0.9608359	0.6413960	0.7740710	1.8673012	0.4301430	3.6430023	0.0134199	5.7162762	-0.0098931
0.0771161	-0.9576662	0.6458660	0.7801289	1.8750005	0.4244750	3.6528417	0.0130379	5.7269993	-0.0098983
0.0784683	-0.9542595	0.6503530	0.7861132	1.8827140	0.4188644	3.6626884	0.0126611	5.7377244	-0.0099044
0.0798337	-0.9506272	0.6548573	0.7920235	1.8904413	0.4133114	3.6725422	0.0122904	5.7484515	-0.0099097

0.0812122	-0.9467798	0.6593787	0.7978595	1.8981827	0.4078158	3.6824033	0.0119250	5.7591806	-0.0099142
0.0826040	-0.9427289	0.6639172	0.8036208	1.9059380	0.4023770	3.6922716	0.0115649	5.7699117	-0.0099196
0.0840091	-0.9384828	0.6684729	0.8093070	1.9137073	0.3969958	3.7021469	0.0112100	5.7806447	-0.0099233
0.0854274	-0.9340504	0.6730457	0.8149178	1.9214904	0.3916711	3.7120294	0.0108598	5.7913797	-0.0099279
0.0868591	-0.9294389	0.6776357	0.8204527	1.9292873	0.3864041	3.7219189	0.0105156	5.8021167	-0.0099316
0.0883041	-0.9246557	0.6822429	0.8259116	1.9370980	0.3811937	3.7318155	0.0101759	5.8128555	-0.0099347
0.0897626	-0.9197081	0.6868672	0.8312938	1.9449226	0.3760401	3.7417190	0.0098407	5.8235962	-0.0099385
0.0912344	-0.9146021	0.6915087	0.8365990	1.9527609	0.3709433	3.7516295	0.0095108	5.8343388	-0.0099415
0.0927197	-0.9093430	0.6961674	0.8418270	1.9606129	0.3659024	3.7615469	0.0091855	5.8450833	-0.0099437
0.0942186	-0.9039361	0.7008432	0.8469773	1.9684786	0.3609183	3.7714713	0.0088655	5.8558296	-0.0099460
0.0957309	-0.8983861	0.7055361	0.8520495	1.9763580	0.3559902	3.7814025	0.0085501	5.8665777	-0.0099483
0.0972568	-0.8926984	0.7102463	0.8570433	1.9842510	0.3511172	3.7913405	0.0082391	5.8773276	-0.0099506
0.0987963	-0.8868768	0.7149735	0.8619583	1.9921576	0.3463003	3.8012854	0.0079327	5.8880793	-0.0099520
0.1003494	-0.8809248	0.7197180	0.8667941	2.0000777	0.3415386	3.8112370	0.0076308	5.8988328	-0.0099536
0.1019162	-0.8748463	0.7244796	0.8715503	2.0080115	0.3368313	3.8211953	0.0073335	5.9095880	-0.0099543
0.1034967	-0.8686453	0.7292584	0.8762265	2.0159587	0.3321794	3.8311604	0.0070407	5.9203449	-0.0099551
0.1050908	-0.8623245	0.7340543	0.8808224	2.0239193	0.3275811	3.8411321	0.0067517	5.9311036	-0.0099558
0.1066988	-0.8558873	0.7388674	0.8853376	2.0318935	0.3230365	3.8511105	0.0064672	5.9418640	-0.0099566
0.1083205	-0.8493362	0.7436976	0.8897716	2.0398810	0.3185464	3.8610955	0.0061866	5.9526260	-0.0099566
0.1099560	-0.8426739	0.7485450	0.8941242	2.0478819	0.3141093	3.8710870	0.0059104	5.9633897	-0.0099566
0.1116054	-0.8359032	0.7534096	0.8983949	2.0558962	0.3097243	3.8810852	0.0056389	5.9741551	-0.0099566
0.1132687	-0.8290265	0.7582913	0.9025833	2.0639237	0.3053931	3.8910898	0.0053710	5.9849220	-0.0099558
0.1149458	-0.8220455	0.7631901	0.9066891	2.0719646	0.3011133	3.9011009	0.0051070	5.9956907	-0.0099551
0.1166370	-0.8149632	0.7681061	0.9107119	2.0800187	0.2968849	3.9111185	0.0048467	6.0064608	-0.0099543
0.1183420	-0.8077818	0.7730392	0.9146512	2.0880860	0.2927089	3.9211425	0.0045903	6.0172326	-0.0099536
0.1200611	-0.8004781	0.7779895	0.9185068	2.0961665	0.2885826	3.9311729	0.0043376	6.0280059	-0.0099520
0.1217942	-0.7930853	0.7829569	0.9222782	2.1042601	0.2845079	3.9412096	0.0040894	6.0387808	-0.0099506
0.1235414	-0.7856044	0.7879415	0.9259650	2.1123669	0.2804830	3.9512527	0.0038443	6.0495572	-0.0099490
0.1253027	-0.7780360	0.7929432	0.9295668	2.1204868	0.2765074	3.9613021	0.0036030	6.0603352	-0.0099468
0.1270781	-0.7703812	0.7979620	0.9330833	2.1286197	0.2725817	3.9713577	0.0033654	6.0711146	-0.0099445
0.1288676	-0.7626410	0.8029979	0.9365141	2.1367657	0.2687044	3.9814196	0.0031309	6.0818955	-0.0099423
0.1306714	-0.7548161	0.8080510	0.9398588	2.1449246	0.2648755	3.9914876	0.0029009	6.0926778	-0.0099399
0.1324893	-0.7469078	0.8131211	0.9431170	2.1530965	0.2610951	4.0015619	0.0026732	6.1034616	-0.0099377
0.1343216	-0.7389168	0.8182084	0.9462884	2.1612813	0.2573623	4.0116423	0.0024500	6.1142469	-0.0099347
0.1361680	-0.7308444	0.8233128	0.9493725	2.1694791	0.2536756	4.0217288	0.0022291	6.1250336	-0.0099316
0.1380288	-0.7226913	0.8284343	0.9523690	2.1776896	0.2500366	4.0318213	0.0020120	6.1358216	-0.0099286
0.1399040	-0.7144588	0.8335729	0.9552776	2.1859130	0.2464430	4.0419200	0.0017986	6.1466110	-0.0099248
0.1417934	-0.7061478	0.8387285	0.9580977	2.1941492	0.2428956	4.0520246	0.0015883	6.1574019	-0.0099218
0.1436974	-0.6977595	0.8439013	0.9608292	2.2023982	0.2393935	4.0621353	0.0013802	6.1681940	-0.0099180
0.1456157	-0.6892949	0.8490911	0.9634716	2.2106599	0.2359353	4.0722519	0.0011768	6.1789875	-0.0099142
0.1475484	-0.6807552	0.8542981	0.9660246	2.2189343	0.2325225	4.0823744	0.0009755	6.1897823	-0.0099097
0.1494957	-0.6721414	0.8595220	0.9684879	2.2272213	0.2291536	4.0925028	0.0007773	6.2005785	-0.0099059
0.1514575	-0.6634548	0.8647631	0.9708609	2.2355210	0.2258279	4.1026371	0.0005821	6.2113759	-0.0099014
0.1534338	-0.6546964	0.8700212	0.9731435	2.2438332	0.2225452	4.1127772	0.0003899	6.2221746	-0.0098969
0.1554247	-0.6458675	0.8752963	0.9753352	2.2521581	0.2193049	4.1229232	0.0002008	6.2329745	-0.0098923
0.1574302	-0.6369692	0.8805886	0.9774358	2.2604954	0.2161070	4.1330749	0.0000146	6.2437757	-0.0098877
0.1594503	-0.6280027	0.8858978	0.9794448	2.2688453	0.2129499	4.1432323	-0.0001684	6.2545781	-0.0098832
0.1614850	-0.6189693	0.8912241	0.9813621	2.2772076	0.2098345	4.1533955	-0.0003492	6.2653818	-0.0098779
0.1635345	-0.6098701	0.8965674	0.9831872	2.2855823	0.2067606	4.1635644	-0.0005270	6.2761866	-0.0098726
0.1655987	-0.6007065	0.9019277	0.9849199	2.2939695	0.2037261	4.1737389	-0.0007018	6.2869926	-0.0098674
0.1676776	-0.5914796	0.9073050	0.9865599	2.3023690	0.2007317	4.1839190	-0.0008742	6.2977998	-0.0098620
0.1697713	-0.5821907	0.9126993	0.9881067	2.3107808	0.1977767	4.1941048	-0.0010437	6.3086081	-0.0098567
0.1718798	-0.5728411	0.9181106	0.9895603	2.3192049	0.1948602	4.2042961	-0.0012109	6.3194176	-0.0098507
0.1740031	-0.5634321	0.9235389	0.9909202	2.3276413	0.1919823	4.2144930	-0.0013751	6.3302282	-0.0098454
0.1761413	-0.5539649	0.9289842	0.9921863	2.3360899	0.1891429	4.2246953	-0.0015370	6.3410399	-0.0098393
0.1782943	-0.5444409	0.9344464	0.9933581	2.3445507	0.1863407	4.2349032	-0.0016966	6.3518527	-0.0098333
0.1804623	-0.5348614	0.9399256	0.9944356	2.3530236	0.1835755	4.2451164	-0.0018540	6.3626666	-0.0098272
0.1826452	-0.5252277	0.9454217	0.9954185	2.3615087	0.1808474	4.2553351	-0.0020083	6.3734816	-0.0098204
0.1848430	-0.5155412	0.9509348	0.9963065	2.3700058	0.1781556	4.2655592	-0.0021604	6.3842976	-0.0098144
0.1870559	-0.5058031	0.9564648	0.9970994	2.3785150	0.1754993	4.2757887	-0.0023094	6.3951147	-0.0098076
0.1892837	-0.4960150	0.9620117	0.9977970	2.3870363	0.1728794	4.2860235	-0.0024570	6.4059327	-0.0098008
0.1915266	-0.4861780	0.9675755	0.9983991	2.3955695	0.1702936	4.2962636	-0.0026022	6.4167518	-0.0097947
0.1937846	-0.4762935	0.9731563	0.9989056	2.4041147	0.1677425	4.3065090	-0.0027445	6.4275719	-0.0097879
0.1960577	-0.4663630	0.9787539	0.9993162	2.4126718	0.1652255	4.3167596	-0.0028851	6.4383929	-0.0097803
0.1983459	-0.4563878	0.9843684	0.9996308	2.4212407	0.1627425	4.3270155	-0.0030236	6.4492150	-0.0097736
0.2006492	-0.4463693	0.9899998	0.9998493	2.4298216	0.1602928	4.3372765	-0.0031598	6.4600379	-0.0097667
0.2029678	-0.4363089	0.9956480	0.9999715	2.4384142	0.1578764	4.3475427	-0.0032929	6.4708619	-0.0097592
0.2053015	-0.4262080	1.0013131	0.9999974	2.4470186	0.1554917	4.3578140	-0.0034254	6.4816867	-0.0097516
0.2076504	-0.4160679	1.0069950	0.9999268	2.4556348	0.1531397	4.3680905	-0.0035547	6.4925124	-0.0097448
0.2100146	-0.4058901	1.0126938	0.9997598	2.4642627	0.1508193	4.3783720	-0.0036818	6.5033391	-0.0097372
0.2123941	-0.3956759	1.0184093	0.9994961	2.4729023	0.1485300	4.3886585	-0.0038074	6.5141666	-0.0097296
0.2147889	-0.3854269	1.0241417	0.9991357	2.4815535	0.1462717	4.3989501	-0.0039315	6.5249950	-0.0097213
0.2171990	-0.3751443	1.0298908	0.9986788	2.4902163	0.1440445	4.4092466	-0.0040525	6.5358243	-0.0097138
0.2196245	-0.3648296	1.0356568	0.9981252	2.4988906	0.1418474	4.4195482	-0.0041720	6.5466544	-0.0097062
0.2220653	-0.3544843	1.0414395	0.9974750	2.5075766	0.1396792	4.4298546	-0.0042901	6.5574854	-0.0096979
0.2245216	-0.3441097	1.0472389	0.9967282	2.5162740	0.1375412	4.4401660	-0.0044059	6.5683171	-0.0096903
0.2269933	-0.3337072	1.0530551	0.9958848	2.5249829	0.1354319	4.4504822	-0.0045193	6.5791497	-0.0096820
0.2294804	-0.3232782	1.0588880	0.9949450	2.5337032	0.1333514	4.4608033	-0.0046313	6.5899830	-0.0096737
0.2319830	-0.3128242	1.0647377	0.9939088	2.5424350	0.1312997	4.4711292	-0.0047410	6.6008172	-0.0096654
0.2345011	-0.3023466	1.0706040	0.9927764	2.5511781	0.1292751	4.4814599	-0.0048499	6.6116521	-0.0096570
0.2370347	-0.2918467	1.0764870	0.9915479	2.5599325	0.1272787	4.4917954	-0.0049559	6.6224877	-0.0096487
0.2395839	-0.2813261	1.0823867	0.9902235	2.5686982	0.1253094	4.5021356	-0.0050610	6.6333241	-0.0096404
0.2421487	-0.2707859	1.0883031	0.9888034	2.5774752	0.1233666	4.5124805	-0.0051639	6.6441612	-0.0096321
0.2447290	-0.2602278	1.0942361	0.9872878	2.5862634	0.1214510	4.5228301	-0.0052652	6.6549991	-0.0096230
0.2473250	-0.2496531	1.1001858	0.9856769	2.5950628	0.1195611	4.5331844	-0.0053651	6.6658376	-0.0096146
0.2499366	-0.2390632	1.1061520	0.9839709	2.6038734	0.1176970	4.5435433	-0.0054627	6.6766768	-0.0096056
0.2525639	-0.2284593	1.1121349	0.9821704	2.6126951	0.1158586	4.5539068	-0.0055596	6.6875167	-0.0095973
0.2552069	-0.2178430	1.1181344	0.9802754	2.6215278	0.1140451	4.5642749	-0.0056541	6.6983573	-0.0095882
0.2578656	-0.2072157	1.1241504	0.9782864	2.6303716	0.1122567	4.5746475	-0.0057472	6.7091985	-0.0095791
0.2605400	-0.1965786	1.1301830	0.9762038	2.6392265	0.1104931	4.5850247	-0.0058387	6.7200404	-0.0095700
0.2632302	-0.1859332	1.1362322	0.9740278	2.6480923	0.1087531	4.5954063	-0.0059288	6.7308829	-0.0095609
0.2659361	-0.1752808	1.1422979	0.9717591	2.6569691	0.1070372	4.6057924	-0.0060173	6.7417259	-0.0095518
0.2686579	-0.1646227	1.1483801	0.9693981	2.6658568	0.1053448	4.6161829	-0.0061043	6.7525696	-0.0095428
0.2713954	-0.1539604	1.1544788	0.9669451	2.6747553	0.1036759	4.6265779	-0.0061897	6.7634139	-0.0095337
0.2741489	-0.1432951	1.1605939	0.9644008	2.6836647	0.1020304	4.6369772	-0.0062738	6.7742588	-0.0095246

0.2769182	-0.1326282	1.1667256	0.9617657	2.6925849	0.1004069	4.6473809	-0.0063570	6.7851042	-0.0095156
0.2797034	-0.1219610	1.1728737	0.9590404	2.7015159	0.0988060	4.6577890	-0.0064379	6.7959502	-0.0095057
0.2825045	-0.1112947	1.1790383	0.9562255	2.7104576	0.0972271	4.6682014	-0.0065174	6.8067968	-0.0094967
0.2853215	-0.1006308	1.1852192	0.9533216	2.7194100	0.0956694	4.6786180	-0.0065960	6.8176438	-0.0094868
0.2881545	-0.0899705	1.1914166	0.9503294	2.7283731	0.0941343	4.6890389	-0.0066732	6.8284914	-0.0094777
0.2910034	-0.0793150	1.1976303	0.9472498	2.7373468	0.0926197	4.6994640	-0.0067489	6.8393395	-0.0094679
0.2938683	-0.0686657	1.2038605	0.9440832	2.7463311	0.0911263	4.7098933	-0.0068230	6.8501881	-0.0094580
0.2967493	-0.0580239	1.2101069	0.9408305	2.7553260	0.0896533	4.7203268	-0.0068964	6.8610371	-0.0094490
0.2996463	-0.0473906	1.2163697	0.9374926	2.7643314	0.0882008	4.7307644	-0.0069682	6.8718867	-0.0094391
0.3025593	-0.0367672	1.2226489	0.9340703	2.7733473	0.0867678	4.7412062	-0.0070386	6.8827367	-0.0094293
0.3054885	-0.0261549	1.2289443	0.9305644	2.7823736	0.0853554	4.7516521	-0.0071074	6.8935872	-0.0094194
0.3084337	-0.0155550	1.2352560	0.9269759	2.7914104	0.0839618	4.7621021	-0.0071756	6.9044381	-0.0094096
0.3113950	-0.0049687	1.2415840	0.9233057	2.8004575	0.0825879	4.7725561	-0.0072429	6.9152894	-0.0093998
0.3143725	0.0056030	1.2479282	0.9195546	2.8095150	0.0812329	4.7830141	-0.0073079	6.9261411	-0.0093899
0.3173661	0.0161587	1.2542886	0.9157239	2.8185828	0.0798969	4.7934761	-0.0073730	6.9369933	-0.0093801
0.3203759	0.0266974	1.2606653	0.9118145	2.8276609	0.0785798	4.8039421	-0.0074358	6.9478458	-0.0093703
0.3234019	0.0372179	1.2670581	0.9078274	2.8367492	0.0772808	4.8144120	-0.0074979	6.9586987	-0.0093604
0.3264441	0.0477190	1.2734672	0.9037638	2.8458478	0.0759992	4.8248859	-0.0075591	6.9695520	-0.0093506
0.3295025	0.0581996	1.2798923	0.8996248	2.8549565	0.0747358	4.8353636	-0.0076189	6.9804056	-0.0093400
